# Molecular Specificity, Convergence and Constraint Shape Adaptive Evolution in Nutrient-Poor Environments

**DOI:** 10.1371/journal.pgen.1004041

**Published:** 2014-01-09

**Authors:** Jungeui Hong, David Gresham

**Affiliations:** Center for Genomics and Systems Biology, Department of Biology, New York University, New York, New York, United States of America; Washington University School of Medicine, United States of America

## Abstract

One of the central goals of evolutionary biology is to explain and predict the molecular basis of adaptive evolution. We studied the evolution of genetic networks in *Saccharomyces cerevisiae* (budding yeast) populations propagated for more than 200 generations in different nitrogen-limiting conditions. We find that rapid adaptive evolution in nitrogen-poor environments is dominated by the *de novo* generation and selection of copy number variants (CNVs), a large fraction of which contain genes encoding specific nitrogen transporters including *PUT4*, *DUR3* and *DAL4*. The large fitness increases associated with these alleles limits the genetic heterogeneity of adapting populations even in environments with multiple nitrogen sources. Complete identification of acquired point mutations, in individual lineages and entire populations, identified heterogeneity at the level of genetic loci but common themes at the level of functional modules, including genes controlling phosphatidylinositol-3-phosphate metabolism and vacuole biogenesis. Adaptive strategies shared with other nutrient-limited environments point to selection of genetic variation in the TORC1 and Ras/PKA signaling pathways as a general mechanism underlying improved growth in nutrient-limited environments. Within a single population we observed the repeated independent selection of a multi-locus genotype, comprised of the functionally related genes *GAT1*, *MEP2* and *LST4*. By studying the fitness of individual alleles, and their combination, as well as the evolutionary history of the evolving population, we find that the order in which these mutations are acquired is constrained by epistasis. The identification of repeatedly selected variation at functionally related loci that interact epistatically suggests that gene network polymorphisms (GNPs) may be a frequent outcome of adaptive evolution. Our results provide insight into the mechanistic basis by which cells adapt to nutrient-limited environments and suggest that knowledge of the selective environment and the regulatory mechanisms important for growth and survival in that environment greatly increase the predictability of adaptive evolution.

## Author Summary

We studied adaptive evolution in different nitrogen-limited environments using long-term selection of asexually reproducing *Saccharomyces cerevisiae* populations in chemostats. Using next generation sequencing and DNA microarrays, we identified all acquired genetic variation associated with increased fitness, in both individual lineages and entire populations. We find that amplification alleles that include nutrient transporter genes specific to the molecular form of the nitrogen present in the environment are a common mechanism underlying increased fitness. In addition, we identified a general strategy for adaptation to nitrogen-limited environments that entails remodeling of phospholipid biogenesis required for producing important cellular components including vacuoles and autophagosomes. More general strategies for adaptation to nutrient-limited environments point to a role for re-wiring of signaling pathways that coordinate cell growth with nutrient availability. We reconstructed the evolutionary dynamics of a population evolving in ammonium-limited conditions and find that a multi-locus genotype is repeatedly selected within the population and constrained by epistasis. We propose that this genotype constitutes a “gene network polymorphism (GNP),” which may be a common outcome of adaptive evolution. Our study suggests that when the selective pressure is understood the molecular basis of adaptive evolution in large microbial populations may be predicted with reasonable precision.

## Introduction

Increasingly, the fields of evolutionary and molecular biology are fusing in a research program that has been termed the “functional synthesis” [1]. The power of this approach is exemplified by the molecular reconstruction of ancestral proteins enabling the study of the functional properties [2] and evolutionary histories [3] of individual genes. By contrast, the evolution of pathways and networks comprising multiple genes has thus far been less amenable to functional studies. This is due in part to the difficulty of inferring and engineering ancestral states of genetic networks. An alternative approach to the study of genetic network evolution is the study of long-term natural selection in laboratories. Experimental evolution using microbes has a number of useful features including the ability to monitor evolution in real time and to measure fitness in the relevant environmental condition [4] that makes it ideally suited to the study of gene network evolution.

Uniquely among experimental methods of long-term selection, continuous culturing using chemostats [5], [6] enables establishment of a precise and invariant selective pressure in which cell growth is continuously constrained by the rate of provision of a growth limiting nutrient. In contrast to evolution experiments using serial dilution [4], [7], [8], in which cells undergo repeated cycles of feast and famine, the unchanging nutrient-poor environment of a chemostat reduces fitness to a single component –continuous growth in a nutrient-poor environment– facilitating testing and interpretation of the functional basis of beneficial mutations. Moreover, in chemostats, large population sizes can be maintained (in excess of a billion cells) during the long-term selection thereby minimizing the effects of genetic drift and population bottlenecks.

Despite recent progress in our understanding of the molecular basis of adaptive evolution in chemostats [9]–[14] many questions remain. Does selection target particular loci and preferentially utilize distinct types of alleles? What is the functional basis of adaptation and are there mechanistic relationships between beneficial mutations? Does increased environmental complexity result in increased heterogeneity within a population? To what extent does epistasis constrain adaptive landscapes? Here, we describe the results of experimental evolution of the budding yeast, *Saccharomyces cerevisiae*, in different nitrogen-limited chemostat environments. Variation in nitrogen availability is frequently encountered in natural ecologies and use of this selection enables comparison with previous adaptive evolution studies in other nutrient-limited environments using chemostats [9], [12], [14].

Importantly, for the goal of understanding genetic network evolution the molecular mechanisms underlying nitrogen utilization in budding yeast have been extensively studied [15], which facilitates interpretation of the functional effects of adaptive mutations. In nitrogen-limited chemostats, the steady-state nitrogen concentration in the culture is extremely low and cells grow continuously in a nitrogen-poor environment. Under these conditions, expression of a set of coordinately regulated genes, the nitrogen catabolite repression (NCR) regulon, is activated by the GATA transcription factors, GLN3 and GAT1 [16]. NCR genes encode a number of transporter and catabolic enzymes for import and assimilation of diverse nitrogen sources, the expression of which is repressed during growth in a nitrogen-rich environment by the negative regulators GZF3 and DAL80 [16].

Despite the greatly simplified and invariant selective conditions of a chemostat we find evidence for at least three distinct adaptive strategies in nitrogen-limited chemostats that operate with different levels of environmental specificity. Consistent with earlier studies in other nutrient limitations [9], [12], [17], comparative analysis among the different nitrogen-limited conditions revealed selection for copy number variant (CNV) alleles that result in increased abundance of transporters specific for the molecular form of nitrogen provided in each environment. We show that these alleles are also selected when multiple nitrogen sources are simultaneously present in the environment and that their inordinate fitness effects likely limit the accumulation of genetic diversity, even in environments with increased environmental complexity. Novel alleles at some loci are recurrently selected in different nitrogen-limited environments, including *VAC14* and genes with related functions, pointing to a role for remodeling of phosphatidylinositol-3-phosphate production and vacuole biogenesis in adaptation to nitrogen-limitation. By integrating our results with previous studies we find that variation in a subset of loci is selected in both nitrogen-limited chemostats and glucose-limited chemostats providing evidence for a general adaptive strategy in nutrient poor environments through remodeling of the TORC1 and Ras/PKA pathways.

We also report a striking example of clonal interference in which independent lineages, defined by mutations in three functionally related loci, *GAT1*, *MEP2* and *LST4* co-evolve in a single population undergoing adaptive evolution in an ammonium-limited chemostat. By studying the individual and interactive effects of these alleles as well as reconstruction of lineage dynamics, we demonstrate that the order of mutations is constrained by epistatic interactions. We propose that this three-locus genotype comprising functionally related gene products represents a gene network polymorphism (GNP), which may be a more frequent outcome of adaptive evolution than previously appreciated.

## Results

To study adaptation in nitrogen-limited environments we founded populations with a haploid *Saccharomyces cerevisiae* strain isogenic to the reference genome (S288c) in different nitrogen-limited chemostats. A normalized concentration of 800 µM nitrogen was used in all feed media making the molecular form of nitrogen the only variable in each environment (Table 1). A single population in each different nitrogen-limited environment was maintained in continuous exponential growth (D = 0.12 culture volumes/hr; t_doubling_ = 5.8 hours) for 250 generations (∼2 months).

**Table 1:** Genetic complexity of adapting populations.

Selective environment (800 µM nitrogen)	Number of SNPs
	(>5% frequency)
Ammonium (400 µM)	10
Arginine (200 µM)	3
Glutamine (400 µM)	1
Proline (800 µM)	11
Glutamate (800 µM)	2
Urea (800 µM)	7
Allantoin (200 µM)	486
Gln/Alla (200/100 µM)	2
Gln/Pro/Alla/Urea (100/200/50/100 µM)	5
Gln/Pro/Alla (133/166/67 µM)	6
Gln/Pro (200/400 µM)	4

A small number of point mutations rose to appreciable frequencies in each population with the exception of the allantoin-limited population, which contains ∼500 SNPs most of which have frequencies less than 10% (see Table S2 and Table S3). This population also contains a mutant *MSH2* gene, suggesting the existence of a low frequency mutator phenotypes [73], [74]. Nitrogen concentrations were normalized between environments by adjusting the concentration of each compound according to its molecular composition.

### Adapted clones have dramatically increased fitness

**Figure 1:**
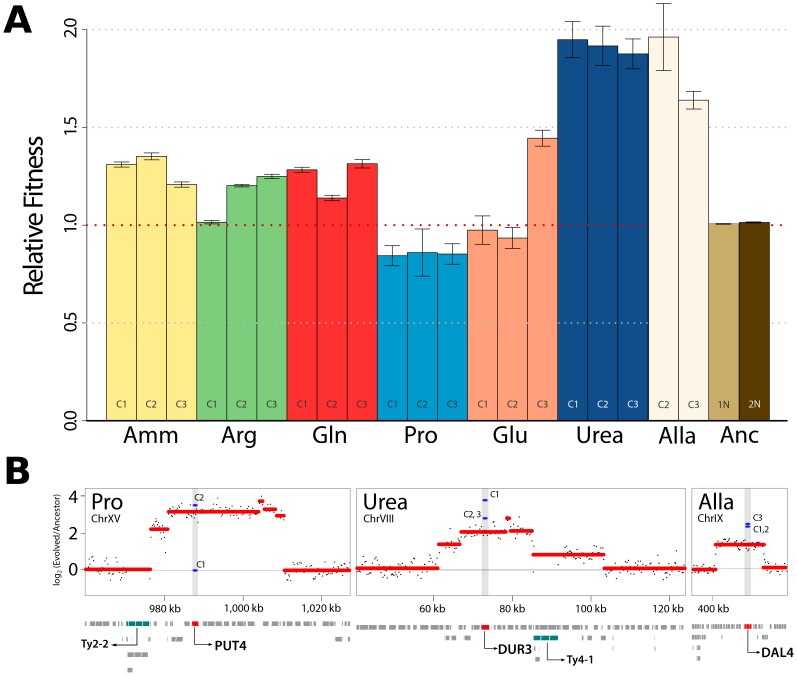
Increased fitness in nutrient-limited environments is associated with amplification of specific permease genes. (A) Fitness increases for clones recovered from each selection are typically >10%. Haploid (1N) and diploid (2N) ancestral strains were also tested in ammonium-limited chemostats but did not show fitness differences. (Amm : ammonium, Arg : arginine, Gln : glutamine, Pro : proline, Glu : glutamate, Urea : urea, Alla : allantoin, Anc : ancestor). (B) DNA copy number was estimated using aCGH. Each black point represents a measurement from a unique probe on the microarray from analysis of population DNA samples. We detected CNVs containing genes with clear connections to nitrogen import at high frequencies in populations (red lines) and clones (blue lines). Retrotransposon (Ty) sequences were frequently found at the boundary regions of CNVs.

Initially, we studied populations evolving in seven different nitrogen-limited environments. To identify phenotypically distinct clones within each adapted population of ∼10^10^ cells following 250 generations of selection we performed batch culture growth rate assays on an unbiased sample of 94 clones from each population and selected three individuals that exhibited growth characteristics distinct from each other and the ancestral strain for further characterization (Figure S1 and methods). We determined the relative fitness of each clone in the appropriate nitrogen-limited chemostat environment and typically observed large increases in fitness (>10%) (Figure 1A). This is consistent with mutation and selection rapidly moving strains towards a fitness optimum. It is clear that the ancestral genotype differs in its distance to the fitness optimum with respect to different nitrogen limited environments: fitness increases in clones selected from ammonium-, arginine- and glutamine-limited chemostats are around 25% whereas fitness increases in clones evolved in urea- and allantoin-limited chemostats exceed 80%. In general, individuals from the same population had similar fitness. A minority of clones did not show increased fitness using this assay for reasons that are not clear, but may be indicative of frequency-dependent selection. The majority of evolved clones were unaltered in their ability to grow in nitrogen-rich conditions or showed decreased fitness (typically less than 4%) (Figure S2). Thus, mutations selected in the nitrogen-poor environments are uniquely beneficial in nitrogen-poor environments and exhibit antagonistic pleiotropy in nitrogen-rich environments.

### Selection for amplification of specific transporter genes

To identify mutations associated with increased fitness we first analyzed the genomes of selected clones, and entire populations, using array comparative genomic hybridization (aCGH). We observed multiple copy number variants (CNVs), including duplicated and deleted genomic regions, typically greater than ∼10 kb, in individual clones and entire populations (Figure S3). Previously, we reported identification of amplification alleles that include the *GAP1* locus in clones adapted to glutamine- or glutamate-limitation [10]. A subset of CNVs present in other nitrogen-limited environments include compelling candidates that are likely to underlie selection of the amplified allele. These include a CNV containing the allantoin permease (*DAL4*) in allantoin-limited conditions, a CNV including the urea permease (*DUR3*) in urea-limited conditions and a CNV including the proline permease (*PUT4*) in proline-limited conditions (Figure 1B). Our ability to detect these CNV alleles in population samples using aCGH (Figure 1B) indicates that they are at high frequency following 250 generations of selection. Consistent with previous studies [9], [18], CNVs are frequently proximal to retrotransposon sequences (Figure 1B), which may increase their spontaneous rate of generation. Previously, we, and others, have identified the repeated selection of copy number variants (CNVs) at the *HXT6/7* [9], [17] and *SUL1* [9] locus in yeast strains selected from glucose- and sulfur-limited chemostats respectively. In *E. coli* evolved in lactulose-limiting conditions the lac operon, which includes the lactose permease (*lacY*), is frequently amplified [19]. Collectively, these findings make clear that in diverse nutrient-limiting conditions, increased production of specific nutrient transporters is a rapid route to increased fitness. The spontaneous rate at which amplification CNVs are generated appears to depend on context [20]; however, estimates of gene amplification rates suggest that they are on the order of nucleotide substitution rates [21]. Selection for spontaneously generated amplification alleles appears to be an expedient means of increasing production of specific nutrient transporters and these alleles are strongly selected in nutrient-poor conditions.

It is notable that we did not detect amplification alleles containing the known high affinity ammonium transporter gene, *MEP2*, in the ammonium-limited population or the arginine transporter, *CAN1*, in the arginine-limited population (Figure S3). It remains to be determined if amplification of *MEP2* or *CAN1* is beneficial in ammonium- or arginine-limited conditions or if these amplification alleles are deleterious for functional or genetic reasons. Moreover, we cannot exclude the possibility that amplification alleles were present at an earlier stage in these populations but were subsequently out-competed.

### Aneuploidy and whole genome duplication may contribute to adaptive evolution

We observed additional copy number variants and entire chromosomal aneuploidies that include genes without obvious connections to growth in nitrogen-limited conditions (Figure S3). We identified 7 aneuploid clones among the 18 analyzed clones (∼40%). The recurrent observation of aneuploidy in adaptive evolution studies [9], [18] and as a mechanism of genetic suppression [22] suggests that they are likely to be adaptive, although the mechanistic basis for the selective advantage of aneuploidies remains to be determined.

We quantified the DNA content of all clones, using flow cytometry, and found that in populations adapted to allantoin- and urea-limitation a high frequency of cells had a 2N DNA content (Figure S4). These individuals are still of a haploid mating type (MATa) as demonstrated by successful mating with MATα cells. The resulting triploid cells underwent sporulation, but typically yielded poor spore viability (<10%) consistent with massive unbalanced chromosome content in the meiotic products of triploids (Figure S4). The maintenance of a MATa mating type in diploid cells recovered from chemostat selections indicates that they are the result of failed cytokinesis and not due to spontaneous mating type switching and subsequent mating. We did not detect a fitness advantage in the chemostat that is attributable to the diploid state *per se* (Figure 1A) consistent with previous studies [23]. Although the high frequency of diploid cells is consistent with selection, the lack of a detectable fitness effect in a wild type diploid cell suggests that selection for diploidization may require the prior acquisition of at least one mutation that is advantageous when increased in copy number as a result of a whole genome duplication.

### mRNA expression levels are correlated with increased copy number at multiple scales

To study the functional basis of adaptation we performed genome-wide transcriptional profiling of evolved clones in the same chemostat environment as they had been selected. Divergence in the transcriptome between clones adapted to different nitrogen environments was qualitatively similar to that seen between clones adapted to glucose- and phosphorous-limited environments [9] (Figure S5). Some of the transcriptional variation in clones adapted to nitrogen-limited environments is a direct result of altered copy number due to CNVs as we detected a small but significant positive correlation between DNA copy number and mRNA abundance (Figure S6A). In general, mRNAs corresponding to transporter genes found within CNVs were increased in abundance, consistent with increased DNA copy number resulting in increased transporter abundance (Figure S6A), providing further evidence that these genes drive selection of the CNV.

As previously observed [24], DNA copy number in disomic or trisomic chromosomes of aneuploid cells is proportional to mRNA abundance level (Figure S6B). In some cases this may explain the selection for a specific aneuploidy. For example, a clone recovered from the glutamine-limitation adaptation contains an additional entire copy of chromosome XI, which contains *GAP1* [10]. However, other chromosomal aneuploidies do not have an obvious connection to nutrient transport making it unclear how, or why, the large-scale increase in expression of genes along duplicated chromosomes of adapted clones contributes to fitness.

### Defining the spectrum of point mutations associated with adaptation

**Figure 2:**
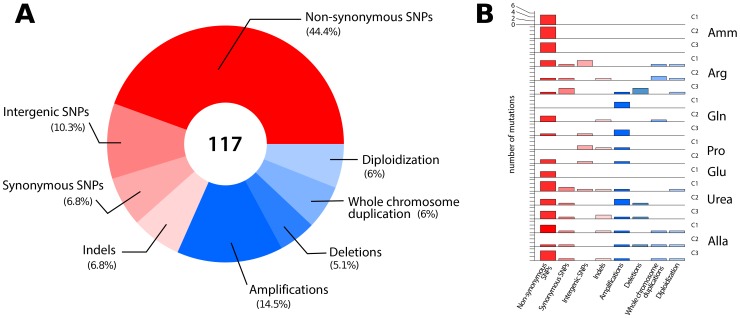
Overview of the classes of mutations identified in lineages adapted to nitrogen-limited conditions. (A) In total, 117 mutational events (Table S1) were identified in 18 sequenced clones resulting in sequence (red) and structural (blue) variation. (B) The number and type of mutations acquired in each individual clones genotyped using aCGH and whole genome sequencing. Non-synonymous SNPs and CNVs are found in most clones.

To identify all mutations acquired during the selection experiments we performed whole genome sequencing of 18 clones from the seven populations (see methods). We found an average of 4 SNPs per clone that together represent a broad range of classes (Figure 2A and Table S1). The average number of SNPs is higher than expected (∼1.0) based on the measured spontaneous nucleotide substitution rate [25] but is consistent with the average number of acquired SNPs (∼3.3) reported for equivalent selections in glucose- or phosphorous-limited environments [13], [26], [27]. Whether this reflects an increased mutation rate under conditions of stress, as reported for *E. coli* [28], or heterogeneity in the number of mitotic events a particular lineage undergoes in a chemostat, remains to be determined. We detected a marginal but statistically significant bias towards SNPs in coding regions: 60/72 SNPs (83%) were found in coding regions, while 72% of yeast genome is coding (exact binomial test, p = 0.035). Although the majority of base changes in coding regions were non-synonymous (52/72; 72%) this is not significantly different than the expected frequency (79%) of non-synonymous mutations [14] (exact binomial test, p = 0.1912). We also identified 8 indels (7 deletions and 1 insertion) of one or two base pairs (Table S1). The average number of indels per clone (∼0.44) is higher than that expected on the basis of the known spontaneous rate of indel events (∼0.06) [25]. All CNVs detected using aCGH were also identified on the basis of sequence read depth. Furthermore, we detected additional deleted genomic segments of several hundred base pairs suggesting that whole genome sequencing has superior sensitivity to aCGH for CNV detection [26] (Table S1). In lineages that had undergone diploidization we detected both homozygous and heterozygous point mutations (Table S1), which allowed us to distinguish mutations that occurred prior to, and after, diploidization, respectively. In sum, comprehensive genome characterization indicates that in individual clones evolving in nitrogen-limited environments, multiple mutations are acquired in a short period of time that range from single nucleotide substitutions to complete duplication of the genome (Figure 2B).

Whereas sequencing of clonal isolates provides information on individual lineages, deep sequencing of entire populations provides a means of assessing the genetic diversity in a population at a particular time point in the evolutionary history of the population [29]. We sought to identify all alleles that had risen to appreciable frequencies following 250 generations of selection using whole genome sequencing of entire populations (Table S2). We identified fixed and non-fixed alleles and estimated their frequencies on the basis of sequence read counts (Figure S7). Despite sequence read depths in excess of 300-fold, we detected few additional mutations in populations that were not identified in clones. Populations typically contained less than 10 SNPs at frequencies >5% (Table 1). A single exception was identified; in the population adapted to allantoin-limitation we found 486 mutations, which is likely the result of mutator phenotype due to loss of function in the mismatch repair gene, *MSH2*, which we estimate to have a frequency of ∼6% in the population (Table S3).

### Increased environmental complexity does not result in increased genetic diversity

We were surprised by the low genetic diversity in populations adapted to individual nitrogen sources (see Table 1) especially since previous analyses of *E. coli* populations evolving in glucose-limited chemostats have suggested the presence of multiple ecotypes [30], [31]. We hypothesized that the low genetic diversity within populations may be a related to the presence of a single nitrogen source in the environment. To study the effect of increasing the complexity of environments on genetic variation in adapting populations, we performed additional long-term selection experiments using mixtures of 2–4 different nitrogen sources. Following the same period of selection we did not detect increased genetic complexity, as assessed by population deep sequencing, in these selections compared with populations adapted to a single nitrogen source (Table 1). We performed aCGH on clones and populations evolved in the presence of mixed nitrogen sources and detected CNVs that include transporter genes specific to individual nitrogen sources present in each environment (Figure S8). However, we did not detect any lineages containing multiple CNVs that would improve transport of more than one of the available nitrogen sources in an environment, suggesting that lineages underwent specialization in the mixed environments. The highest frequency CNVs in populations adapted to mixed nitrogen sources transport non-preferred nitrogen sources (proline, allantoin and urea) (Figure S8), which also tend to be associated with the greatest individual fitness increases (Figure 1A). Collectively, our observations in single and mixed nitrogen-limited environments are consistent with a highly skewed distribution of fitness effects in which CNV alleles that include transporter genes have large fitness effects and therefore a high probability of sweeping to fixation. The large effect sizes of these CNV alleles limits genetic diversity even in environments of increased complexity.

### Identification of specific and convergent targets of selection

**Figure 3:**
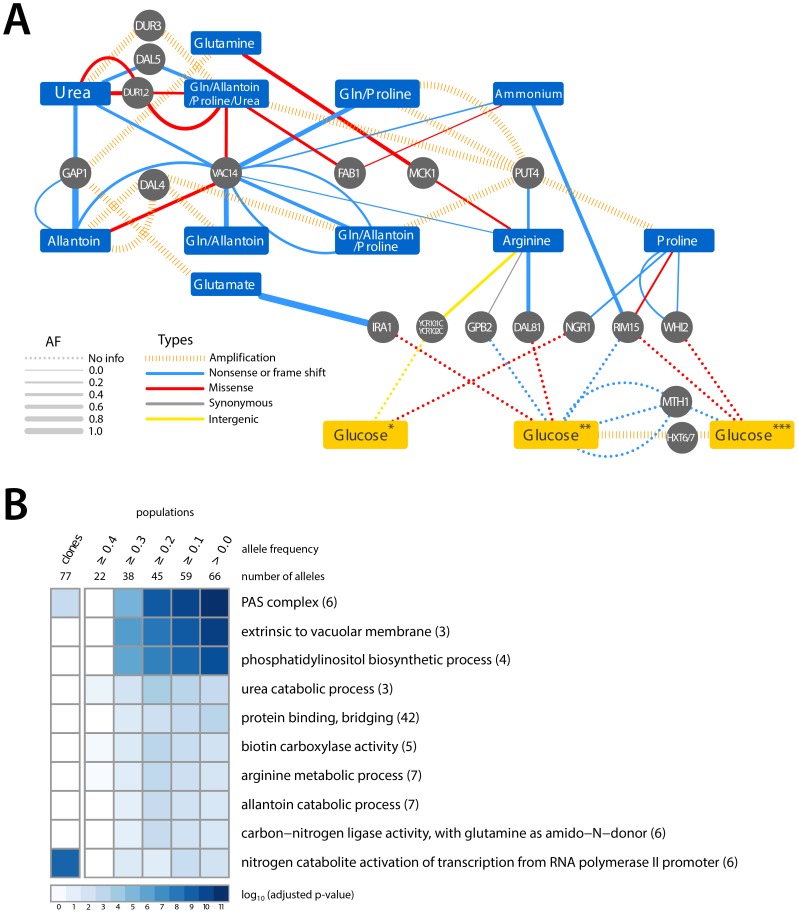
Adaptive mutations occur in functionally related loci. (A) A small number of loci are mutated in multiple nitrogen-limited environments. Some loci found to be mutated in nitrogen-limiting conditions have also been reported as associated with adaptive evolution in glucose-limited environments (*Wenger, J. et al [14], **Kvitek, D.J. et al [13], ***Gresham, D. et al [9]). The color of edges represents the type of allele and the width of the edge represents the frequency of the allele in the population. (B) GO term enrichment analysis of mutated loci within clones and populations, analyzed at different allele frequency thresholds, identified in nitrogen-limited environments shows enrichment for specific cellular functions.

High throughput sequencing of clones and populations revealed that genetic variation at a number of loci was repeatedly selected in different nitrogen-limited selections (Figure 3A). In addition to amplification of permease genes in conditions in which they increase import rates of nitrogen-containing compounds, we find that inactivating alleles are selected in conditions in which their function provides no benefit or may be deleterious. As we previously reported, this is the case for *GAP1*, which is amplified in glutamine- and glutamate-limited conditions and deleted when the nitrogen source is not an amino acid such as allantoin and urea [10] (Figure 3A). Similarly, amplification alleles containing *PUT4*, which encodes a proline permease, are selected in environments in which proline is a nitrogen source, but an inactivating mutation in *PUT4* was found in the arginine-limited environment. We hypothesize that loss of function mutations in these genes are selected as the NCR-derepressing conditions of a nitrogen-limited chemostat result in their high expression, which is futile in the absence of the substrate(s) they transport.

We identified six loci that acquired point mutations in multiple nitrogen-limitation selections. The most striking of these was *VAC14*, which is mutant in 8 of the 11 different selective environments. Sequence variants in *VAC14* are predominantly loss of function mutations and in two populations we found multiple independent *VAC14* alleles (Figure 3A). *VAC14* encodes a scaffold component of the protein complex regulating inter-conversion of phosphatidylinositide-3-phosphate (PI3P) to phosphatidylinositide-3,5-bisphosphate (PI(3,5)P_2_) [32]. Interestingly, an additional repeatedly mutated locus, *FAB1*, encodes the 1-phosphatidylinositol-3-phosphate 5-kinase that functionally interacts with VAC14. When all mutations identified in clones and populations are considered (Table S1 and Table S2), there is a clear enrichment for molecular functions related to phosphatidylinositol biosynthetic processes and the related processes of autophagosome and vacuole biogenesis (Figure 3B) indicating that they are a convergent target of selection across nitrogen-poor environments. Functional enrichment analysis of mutations in populations and among clones also identified several additional molecular processes related to nitrogen metabolism (Figure 3B). Thus, the molecular basis of adaptive evolution in nitrogen-limited environments exhibits convergence at both the level of individual genes, and at the level of modules, defined by functionally related genes.

It is possible that some adaptive alleles recovered in our experiments are not specifically related to nitrogen utilization, but underlie adaptation to the requirement of continuous growth in nutrient-limited conditions. To identify such loci we compared the loci associated with adaptive evolution in nitrogen-limited environments with those identified in previous studies of adaptation to glucose-, phosphate- and sulfur-limited environments [9], [12]–[14] (Figure 3A). Several loci mutated in both glucose- and nitrogen-limited chemostats encode components of signaling pathways that regulate cell growth in response to the nutritional state of the environment. At least two of these genes (*RIM15* and *WHI2*) regulate entry into a quiescent (G_0_) state. Loss of the ability to enter G_0_ may be beneficial in the chemostat, as even transient entry into G_0_ will prolong the cell division cycle leading to cells being outcompeted. Selection for this class of mutations may be analogous to the recurrent loss of function mutations found in the stress response sigma factor, *rpoS*, in experimental evolution of *E. coli* in chemostats [33]. No mutated loci were shared with phosphate and sulfur-limited selections.

### Identification of a recurrently selected three-locus genotype comprising functionally related genes

**Figure 4:**
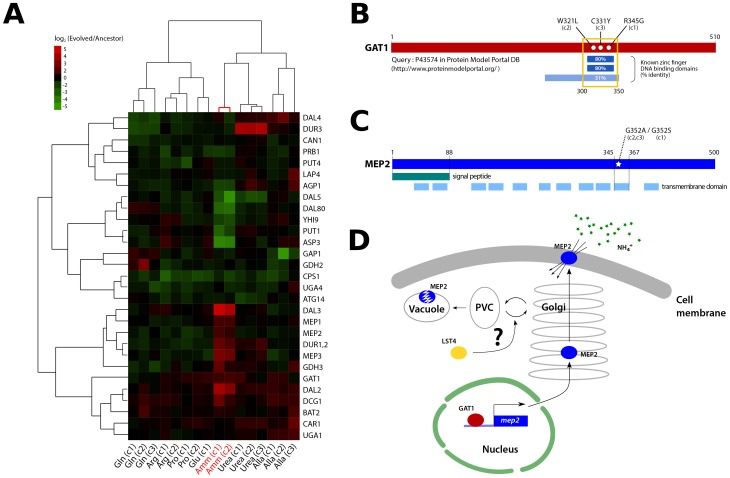
Functional effects of adaptive mutations in a gene network polymorphism. (A) NCR genes are altered in expression in clones recovered from ammonium-limited conditions. Only genes having at least one observation with log_2_ ratio >|1.5| were included (29/38 NCR genes [71]). Genes and samples are hierarchically clustered using centered correlation and complete linkage. (B) Three independently acquired *GAT1* mutations found in a single ammonium-limitation adapted population are clustered in the zinc finger DNA binding domain of the encoded protein. The wild type GAT1 protein sequence was queried using the Protein Model Portal database [72]. (C) Two different point mutations in MEP2 found in ammonium-limitation adapted clones change the identical codon within a putative trans-membrane domain. Domain information was obtained from SGD database (http://www.yeastgenome.org/). (D) GAT1 and LST4 likely regulate the production and delivery of MEP2 to the plasma membrane at the transcriptional and post-translational level, respectively.

The population adapted to ammonium-limitation was the only population in which we did not detect evidence of CNVs in either clones or the entire population (Figure 2B, Table S1 and Table S2). However, clones from this population displayed the greatest divergence in nitrogen catabolite repression (NCR) gene expression among all clones analyzed (Figure 4A and Figure S9) and had large fitness increases (Figure 1A) suggesting that they had undergone significant adaptive evolution.

We found that these two clones, and a third that was not analyzed for gene expression, contain mutations in the DNA binding domain of the zinc finger transcription factor *GAT1* (Figure 4B), which encodes a positive regulator of NCR expression [15]. A subset of NCR genes is increased in expression in these clones including those encoding the high affinity (*MEP2*) and low affinity (*MEP1* and *MEP3*) ammonium permease genes (Figure 4A). Interestingly, several NCR transcripts are also decreased in expression suggesting that the *GAT1* mutations may have differential effects on its transcriptional targets.

In addition to mutations in *GAT1*, we found that the three clones from the ammonium-limitation selection contained one of two different mutations in the identical codon of a predicted transmembrane domain of the high affinity ammonium transporter *MEP2*, a transcriptional target of GAT1 [34] (Figure 4C). Furthermore, two of these clones contained mutations in *LST4*, which encodes a protein required for efficient sorting of permeases from the Golgi to plasma membrane [35]. The acquired mutations in *LST4* are unlikely to render it non-functional based on drug sensitivity assays (Figure S10). The three genes, *GAT1*, *MEP2* and *LST4* that comprise this recurrently selected multilocus genotype encode functionally related gene products (Figure 4D) consistent with adaptive evolution proceeding via the sequential accumulation of variation in genetic networks within lineages.

### Population dynamics of the three-locus genotype

**Figure 5:**
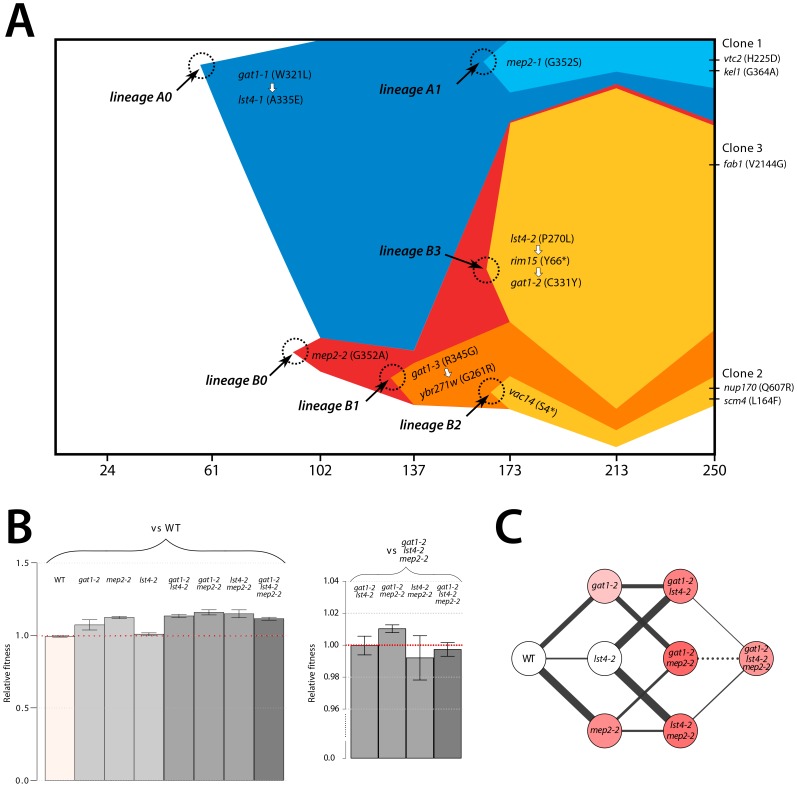
Recurrent selection and evolutionary dynamics of a GNP. (A) Estimated genotype dynamics during adaptive evolution. The time of introduction of each new mutation (dotted circles) is estimated on the basis of detecting an allele frequency of at least 5% in the population. Some mutations were clustered based on their similarity in the dynamics (see Figure S11). The temporal order of mutations that occurred in rapid succession (white arrows) was determined on the basis of their allele frequencies in the final evolved population estimated using deep sequencing data (Figure S7). (B) Fitness estimates of 8 backcrossed strains, representing all possible combinations of alleles that comprise the GNP, from clone 3 isolated from the ammonium-limitation selection were determined by direct competition with either the ancestral or the *gat1-2*/*lst4-2*/*mep2-2* genotypes. Error bars are 95% CI of the regression coefficient. (C) Fitness landscape reconstruction based on the fitness estimates for the 8 genotypes. The selection coefficient values of each strain are represented as color intensity. The width of each edge is proportional to the difference in fitness between two genotypes that edges connect. A solid line indicates a favored path whereas a dashed line indicates a disfavored path. Selection favors thicker, solid lines in the evolutionary trajectory.

We aimed to determine the temporal dynamics with which the mutations in *GAT1*, *MEP2* and *LST4* occurred and were selected. Population sequencing of the ammonium-limitation adapted population after 250 generations of selection identified 10 SNPs with detectable allele frequencies (>5%) (Table S1 and Table S2). Allele frequencies in the population are informative about the order in which mutations were acquired in each asexually reproducing lineage; however, the timing of mutational events cannot be deduced on the basis of allele frequencies. To reconstruct the evolutionary history of the lineages we determined allele frequencies throughout the evolution experiments using Sanger sequencing [9] (methods; Figure S11). The resulting trajectories (Figure 5A) show that within a single population the same two locus genotype (*gat1*, *mep2*) was independently generated and selected three times (lineages A1, B1, and B3) and the three locus genotype (*gat1, mep2, lst4*) was generated at least twice (lineages A1 and B3). Interestingly, in both lineages, mutations in *GAT1* and *LST4* occurred in rapid succession and subsequently increased in frequency (i.e. lineage A0 and lineage B3 in Figure 5A), which is suggestive of a synergistic interaction between *LST4* and *GAT1*. Although we detect dramatic changes in allele frequencies during the selection no individual genotype swept to complete fixation (i.e. a “hard sweep”). Rather, competition (i.e. clonal interference) between lineages bearing different alleles in the identical multi-locus genotype resulted in alternating “soft sweeps”.

### Epistasis constrains the order of mutational events

As functionally related genes are enriched for genetic interactions [36], we hypothesized that epistatic interactions might exist between *GAT1*, *MEP2* and, *LST4*. To test this hypothesis we constructed strains containing the eight possible combinations of the *gat1-2*, *lst4-2* and *mep2-2* alleles identified in clone 3 (methods). The mutations in *MEP2* and *GAT1* are individually beneficial; however, the mutation in *LST4* does not confer a selective advantage on its own (Figure 5B). The double mutation genotypes comprised of either *mep2-2* and *lst4-4* or *gat1-2* and *lst4-2* are more fit than expected by summation of their individual fitness effect providing evidence for positive epistasis. However, we found that the combined effect of the *gat1*-2/*lst4*-2/*mep2*-2 alleles does not result in significantly increased fitness compared with the *gat1-2*/*lst4-2* or *mep2-2*/*lst4-2* double mutant genotypes consistent with negative epistasis. To more accurately compare fitness effects of different genotypes we directly competed double mutant genotypes directly with the *gat1*-2/*lst4*-2/*mep2*-2 genotype. Consistent with our initial observations we find that the *gat1*-2/*lst4*-2/*mep2*-2 triple mutant genotype is not significantly fitter than the *gat1*-2/*lst4*-2 or *lst4*-2/*mep2*-2 double mutant genotypes and is in fact significantly less fit than the *gat1*-2/*mep2*-2 genotype. Thus, an *LST4* mutation is beneficial only in the background of an individual mutation in *GAT1* or *MEP2* whereas it is detrimental in the background of the *GAT1*/*MEP2* double mutant (Figure 5C). This sign epistatic interaction is consistent with the order of mutation acquisition in the three lineages in the population: an *LST4* mutation is observed after the occurrence of a *GAT1* mutation (lineage A0) or a *MEP2* mutation (lineage B3), but not in the lineage that contains a mutation in both *GAT1* and *MEP2* (lineage B1).

## Discussion

A major motivation for Novick and Szilard's introduction of the chemostat was the study of spontaneous mutations and evolution [5]. Seminal studies by Paquin and Adams in the 1980s established the use of budding yeast in experimental evolution studies in chemostats [37], [38]. The advent of genome-scale methods for comprehensive identification of changes in gene expression [39], structural genomic variation [18] and DNA sequence [40] provided insight into the molecular basis of adaptive evolution in chemostats. For many years, experimental evolution using chemostats and budding yeast have primarily been performed using glucose as the growth limiting substrate. More recently, we reported a survey of adaptive evolution of budding yeast in glucose-, phosphate- and sulfur-limited environments [9]. Comparison among these selections revealed that the number of adaptive strategies differs as a function of the selective pressure and thus the details of the selective regime dictate the “repeatability” of evolution. Here, we have built on our recent report of adaptation in nitrogen-limited chemostats [10] to yield a comprehensive survey of adaptive strategies in environments that are limited for different sources of nitrogen. Our new study allows us to draw several general conclusions about the mechanistic bases of adaptive evolution in nutrient-poor environments and provide new insight in the complexity and dynamics of adaptive evolution.

### Alleles that specifically increase the transport kinetics of the compound containing the growth-limiting nutrient are recurrently selected

In a chemostat, the rate of cell growth is constrained by the concentration of a single nutrient that is essential for growth [41]. Thus, there is intense selective pressure for adaptive strategies that improve the import or metabolism of the growth-limiting nutrient. In our study, we initially provided a single source of nitrogen at a growth-limiting concentration. We observed massively increased fitness of in selected lineages following 250 generations of selection when fitness was assessed in the same environment as that in which the selection was performed. In the majority of cases, analysis of individual lineages identified CNVs that include a transporter gene that specifically transports the molecular form of nitrogen provided in the environment. Thus, in addition to the amplification of the *GAP1* locus in glutamine- and glutamate-limited conditions [10], we find *DUR3* amplification alleles in urea-limited environments, *DAL4* amplification alleles in allantoin-limited environments and *PUT4* amplification alleles in proline-limited environments. The fact that these CNVs are detected in DNA samples of entire populations indicates that they are at high frequency in these populations, most likely as a result of selection. Transcriptome analysis indicates that these alleles result in increased gene expression, which likely results in increased protein production. Our new results are consistent with previous studies in budding yeast that have identified amplification of the *HXT6/7* locus in populations adapted to glucose-limitation [9], [12], [17] and amplification of the *SUL1* locus, encoding the high affinity sulfur-permease, in populations adapted to sulfur limitation [9]. The large fitness increases attributable to these specific CNV alleles means that they dominate the evolutionary dynamics of adapting populations thereby limiting the genetic diversity in nutrient-limited environments. CNV alleles have been reported to underlie increased fitness in a diversity of selective environments and organisms, including humans, suggesting that they are a class of genetic variation that are of general importance for adaptive evolution.

Increased fitness associated with nutrient transporter amplification is specific to nutrient-poor environments. Using competitive growth rate assays in nitrogen-rich environment we find that evolved clones tend to have decreased fitness. Similar fitness trade-offs in carbon-rich environments have been reported for lineages adapted to glucose-limited chemostats [14]. Amplified transporter alleles may be an underlying source of this antagonistic pleiotropy. Previously, we have shown that inactivating mutations in *GAP1* are selected in chemostats containing limiting concentrations of non-amino acid nitrogen sources [10]. In the current study we identified a *PUT4* inactivating mutation in a lineage evolved under arginine limitation (Figure 3A). In environments in which the limiting nutrient is present in a predominant molecular form, loss of some transporter genes may be beneficial either through reduction in the energetic cost of their unnecessary production or as a result of a function that is deleterious in the particular environment. Future work will be required to rigorously test the hypothesis that CNV alleles are a molecular basis of antagonistic pleiotropy.

### A hierarchy of generalist strategies underlies adaptive evolution in nutrient-poor environments

In addition to selection of specific transporter amplification alleles in different nitrogen-limited environments, we find evidence for convergent routes to increased fitness across different nitrogen-limited environments. The most striking evidence comes from the multiple inactivating and nonsynonymous mutations that we identified in *VAC14*. We found at least one, and as many as three, independent alleles within the 2.6 kb coding region of *VAC14* in eight of the eleven populations that we studied (Figure 3A). *VAC14* encodes a scaffold component of the protein complex regulating inter-conversion of phosphatidylinositide-3-phosphate (PI3P) to phosphatidylinositide-3,5-bisphosphate (PI(3,5)P_2_) [32]. In addition, we found mutations in *FAB1*, which encodes a PI3P 5-kinase and *VAC7*, a regulator of FAB1, in different nitrogen-limited populations, albeit, much less frequently than *VAC14* mutations (Table S1). Control of PI(3,5)P_2_ levels by VAC14, VAC7 and FAB1 is important for several cellular processes including protein trafficking and maintenance of vacuole size and acidity [42], [43]. Loss of function of VAC14 results in decreased PI(3,5)P_2_ levels leading to enlarged vacuoles due to defective vacuolar fission [44]. Enlarged vacuoles may be beneficial in nitrogen-limited conditions as vacuoles function as a reserve for nitrogen stores as well as being the compartment for recycling of cytosolic proteins through autophagy [45]. Non-synonymous mutations in the *VAC7* and *FAB1* may have similar consequences on PI(3,5)P_2_ levels and vacuole biogenesis as VAC14 loss of function mutations. Although identifying the precise mechanistic basis by which mutations in these functionally related genes contribute to increased fitness in nitrogen-limited environments requires additional study, their selection in different nitrogen-limited environments, and their absence in the mutational spectra identified in other nutrient-limited conditions reported to date, suggests that novel alleles at these loci underlie a generalist strategy specific to nitrogen-limited conditions.

By integration of our results with previous studies in other nutrient-limited environments, we find evidence for adaptive strategies involving remodeling of the TORC1 and Ras/PKA signaling pathways that may be general to nutrient limitation. These signaling pathways control cellular growth rate in response to nutrient availability by regulating diverse cellular processes [46], [47]. In particular, mutations in the regulator of cell cycle exit and entry into G_0_, *RIM15* are found in different glucose- and nitrogen-limitation selections (Figure 3A). RIM15 is known to have an important role in integrating signals from multiple nutrient responsive signaling pathways including TORC1 and Ras/PKA [48], [49]. A reduced capacity to enter a G_0_ state could be beneficial in a variety of nutrient-limitations in chemostats. Consistent with this hypothesis, additional genes that are mutant in both nitrogen- and glucose-limited chemostats include *WHI2*, a negative regulator of G_1_ cyclin expression, *IRA1* and *GPB2*, both of which are negative regulators of the Ras/PKA pathway, and *NGR1*, an RNA-binding protein involved in regulation of cell growth control. Selection for this class of mutations in different nutrient limitations is consistent with the argument that recurrent selection for loss of *rpoS* in *E. coli* populations evolved in glucose-, nitrogen- [50] and phosphorous-limited [51] chemostats underlies a tradeoff between the cellular response to nutrient starvation and maintenance of stress resistance.

### Selected variation accumulates in genetic networks under epistatic constraints

Although transporter amplifications dominate the majority of our adaptive evolution experiments, we did not identify transporter amplification alleles in two of our populations (ammonium and arginine limitation); the population that underwent adaptive evolution in an ammonium-limited environment was the only population in which we did not identify any CNVs or large-scale chromosomal events. Nutrient transport is still a primary target of selection in this population as we found two independently acquired non-synonymous SNPs that result in amino acid substitutions at the same amino acid residue in MEP2 (G352A and G352S). The mutated site is in a predicted trans-membrane domain (Figure 4C) making it likely that these mutations alter the affinity of MEP2 for ammonium either directly or indirectly. Fitness tests of one of a strain containing one of these mutations (G352A) show that this variant confers a fitness increase exceeding 10% (Figure 5B). Interestingly, we find evidence that independently generated alleles containing this precise variant may have been selected in natural yeast populations. Although our ancestral strain, which is isogenic to S288c, encodes a glycine at residue 352 in MEP2, this site is polymorphic among *S. cerevisiae* strains with 19/26 strains in the SGD database (http://www.yeastgenome.org) encoding an alanine at residue 352. Moreover, the reference genomes of *Saccharomyces sensu stricto* species, including *S. uvarum*, *S. mikatae*, and *S. paradoxus*, all contain an alanine at residue 352 in MEP2 homologues. It is interesting to note that a recent study reported recurrent selection of *MEP2* fusion alleles when a hybrid *S. cerevisiae*/*S. uvarum* strain was evolved in ammonium-limited chemostats [52]. *S. cerevisiae* and *S. uvarum* differ at 17 residues in the MEP2 protein, one of which is the 352nd amino acid. Consistent with the importance of the 352A allele under conditions of ammonium-limitation, all independently selected *S. cerevisiae*/*S. uvarum MEP2* fusion alleles retained the carboxy terminus-encoding portion of the *S. uvarum MEP2* allele, which codes for an alanine at codon 352. Collectively, these observations suggest that the selection that we imposed in the laboratory bears some resemblance to selection experienced by yeast cells in the natural world with a strikingly convergent response to selection at the molecular level.

The population adapted to ammonium-limitation provides evidence that accumulation of variation in functionally related genes underlies adaptive evolution in nutrient-limited environments. Two lineages within the population that contain mutations in *MEP2* also contained mutations in *GAT1*, which encodes a transcriptional activator of *MEP2* (in addition to other NCR genes) as well as mutations in *LST4*, which encodes a protein that functions in protein sorting to plasma membranes [53]. Analysis of the dynamics with which these mutations were selected demonstrates that their sequential acquisition underlies clonal interference dynamics in this population. Clonal interference due to multiple independent mutations at the same locus has been documented in a variety of experimental evolution studies (e.g. [54]). Our current results show that competing lineages in the same population can accumulate mutations at multiple, common loci as has been observed in *E. coli* [29]. Interestingly, unlike the recurrently selected three locus genotype identified in [29] comprising variants in *spoT*, *rbs* and *nadR*, which encode functionally unrelated gene products the three loci that define the recurrently selected genotype identified in our study, *GAT1*, *MEP2* and *LST4*, comprise a functionally related gene network (Figure 4D).

The order in which mutations at these three loci are acquired appears to be constrained by epistatic interactions. By studying all possible allelic combinations at these three loci we determined that the *lst4-2* allele exhibits positive epistasis with the *mep2-2* and *gat1-2* alleles individually. However, the two locus *gat1-2*/*mep2-2* genotype is more fit than the three locus *gat1-2*/*mep2-2/lst4-2* genotype (Figure 5C). This negative epistatic interaction is consistent with the observation that an *LST4* mutation occurs in the background of a *GAT1* mutation (lineage A0) or a *MEP2* mutation (lineage B3), but does not occur in the lineage in which both a *GAT1* and *MEP2* mutation has already occurred (lineages B1 and B2) (Figure 5A). It is also interesting to note that the double mutant genotypes (*gat1-2*/*lst4-2* and *lst4-2*/*mep2-2*) and the triple mutant genotype (*gat1-2*/*lst4-2*/*mep2-2*) do not differ significantly in their fitness (Figure 5C), suggesting that they will coexist in an evolving population. Consistent with this expectation, the lineages A0 and A1, which differ only at LST4 and the lineages B1 and B3, which differ at LST4 and two additional loci, co-exist that for around 100 generations (Figure 5A).

Increasingly, resolution of the multigenic basis of quantitative trait variation to nucleotide variants demonstrates that allelic variants in functionally related genes underlies adaptive evolution [55], [56]. As the multi locus genotype that we have identified is 1) comprised of functionally related gene products that 2) interact epistatically with one another, we propose that it comprises a gene network polymorphism (GNP) similar to that reported for the galactose-utilization regulon segregating in diverged *Saccharomyces kudriavzevii* populations [57]. Given a sufficiently large population size, we show that nearly identical GNPs can be recurrently generated and selected within a population resulting in “soft sweeps” in which the GNPs are maintained at intermediate frequencies. The rapid generation of a GNP in a particular niche may lead to balanced unlinked GNPs (buGNPs) segregating in the larger population as observed in the *Saccharomyces kudriavzevii* population [57].

### Conclusion

Our study provides new insight into the functional basis of adaptive evolution in nutrient-limited environments. Consistent with the low concentration of a single growth-limiting substrate representing the dominant selective pressure in a chemostat we find evidence for strong selection of alleles that enhance transport of the specific molecular form of the limiting nutrient. In addition, we have identified a mechanism underlying adaptive evolution that appears to be shared among different nitrogen-limited environments, involving phospholipid metabolism and vacuole biogenesis, and a mechanism shared between nitrogen- and carbon-limited environments, entailing nutrient-responsive growth regulating pathways. The identification of a finite number of adaptive strategies in nutrient-limited environments suggests that adaptive evolution of large populations in nutrient-limited environments proceeds along a limited number of paths. Thus, the combination of precise knowledge of the selective environment experienced by a population of organisms and the molecular mechanisms that underlie growth and survival in that environment is likely to greatly enhance the predictability of adaptive evolution.

## Materials and Methods

### Strains and media

For all adaptive evolution experiments we founded populations with a haploid derivative (FY4) of the S288c reference strain. For competition assays, we integrated constitutively expressed mCherry or mCitrine-labeled constructs, marked with the kanMX4 cassette, at the HO locus using the high efficiency yeast transformation protocol [58]. All nitrogen-limiting media contained 800 µM nitrogen regardless of the molecular form of the nitrogen and 1 g/L CaCl_2_-2H_2_O, 1 g/L of NaCl, 5 g/L of MgSO_4_-7H_2_O, 10 g/L KH_2_PO_4_, 2% glucose and trace metals and vitamins as previously described [59].

### Long-term selection

We founded populations with FY4 in 200 mL of nitrogen-limited media. Chemostat cultures were maintained using Sixfors fermentors (Infors) at 30°C, constantly stirred at 400 rpm in aerobic conditions and diluted at a rate of 0.12 hr^−1^ (population doubling time 5.8 hr). Each steady-state population of ∼10^10^ cells was maintained in continuous mode for 250 generations (∼2 months). A 2 mL population sample was obtained every 20 generations and archived at −80°C in 15% glycerol.

### Isolation of clones

Following 250 generations of selection we randomly plated cells onto rich media (YPD), and selected an unbiased sample of 94 clones. We grew all clones from each population in 96 well plates containing the same nitrogen source as that used in the selection experiment and recorded optical densities at 600 nm every 0.5 hr over 24 hours using a 96-well Tecan plate reader. Each plate included the ancestral strain (FY4) and a blank well. We estimated the growth rate and the saturation density of all strains using the ‘*grofit*’ package [60] in R and selected three clones from each population for further analysis.

### Determination of cell ploidy

We determined the DNA content of evolved clones by staining with Sytox green and analyzing at least 10,000 cells using flow cytometry. FY4 and an isogenic diploid (FY4/FY5) were used for calibration. In addition, each evolved clone was mated with an isogenic strain (FY5) of the opposite mating type (MATα). The resulting strain was sporulated and at least 20 tetrads were dissected using a micromanipulator. Spore viability was determined after three days growth on YPD at 30°C.

### Fitness estimates

Each mutant was competed in a chemostat against the ancestral strain (FY4) or a mutant bearing *gat1-2*, *mep2-2*, and *lst4-2* mutations, engineered to constitutively express either mCherry or mCitrine, in the same nitrogen-limited condition used in the selection experiment. We inoculated the unlabeled evolved clone and labeled reference strain in separate chemostat vessels and obtained steady-state cultures of 200 mL. We then mixed the evolved clone with the labeled reference strain to a final ratio of 1∶5. We obtained 2 mL samples every 2–3 generations over a total of ∼20 generations. Samples were stored at 4°C in phosphate buffered saline (PBS) containing 0.01% Tween 20. The relative ratio of the fluorescently labeled reference strain and the unlabeled evolved clone was measured by counting at least 100,000 cells from each sample using flow cytometry. We used linear regression of the log transformed (ln) ratio of evolved/reference strain abundance against time (in generations) to estimate the selection coefficient (*s*, the slope of the fit linear line) and associated standard error (*s.e*) using the ‘*lm*’ function in R. We calculated the 95% confidence interval of the regression coefficient in R. The relative fitness, normalized to wild type, is *1+s*. Competition assays in batch culture were performed using synthetic deficient (SD) media containing 5 g/L ammonium sulfate and were performed using analogous methods by first growing evolved and fluorescently-labeled ancestral strains in isolation to log phase and then mixing them at a 1∶1 ratio. Cultures were maintained in log phase growth for 24 hours (less than 12 generations) and sampled 5–6 times. The relative abundance of the two strains and fitness coefficients were determined using the same flow cytometry and analytical methods used for chemostat competitions.

### DNA microarrays

RNA samples were obtained from evolved clones grown in chemostats limited for the same nitrogen source in which they had been selected. In addition, we obtained RNA samples of the ancestral strain (FY4) grown in each of the nitrogen-limited conditions. Gene expression profiling was performed using Agilent 60-mer DNA microarrays as previously described [9], [24]. We used a common reference for all expression analysis, obtained from a sample of the ancestral strain grown in an ammonium sulfate-limited chemostat growing at a dilution rate of 0.12 hr^−1^. We identified gene expression variation specific to evolved clones by normalizing each mRNA abundance measurement with the expression level of that transcript in the ancestral strain grown in the same environment.

Array Comparative Genomic Hybridization (aCGH) was performed using Agilent 60mer DNA microarrays as previously described [9], [24]. Genomic DNA (gDNA) from evolved clones and entire populations was prepared using the QIAGEN genomic DNA extraction kit, labeled with Cy3 and co-hybridized with Cy5-labeled DNA from the ancestral strain. The resulting log_2_ transformed ratio was segmented using the ‘*DNAcopy*’ package [61] in R.

### Library preparation for next-generation sequencing

We obtained gDNA from each evolved clone and the ancestral strain (FY4) from 10 mL overnight cultures using the QIAGEN genomic DNA extraction kit. For population samples, gDNA was extracted from 10 mL samples taken directly from the adapting population. 1 µg of gDNA sample was then sonicated in a Covaris AFA to obtain fragments of 300–500 bp. To blunt the ends of fragmented gDNA we incubated with PNK (10 Unit) and T4 DNA polymerase (12 unit) at 20°C for 30 min, and then purified using QIAGEN Min-Elute Columns. Adenosine overhangs were added to the blunted DNA using Exo(-) Klenow (15 Unit) incubated at 37°C for 20 minutes, followed by purification using QIAGEN Min-Elute Column and elution in 19 µL EB buffer. To multiplex genome sequencing we ligated one of six unique 120 bp adapters (BIOO) using Quick ligase at 23°C for 20 minutes. The ligated samples were purified, and adaptor dimers removed, using AMPure XP beads (Agencourt). The purified samples were loaded on a 2% agarose gel with TAE buffer, run at 100 V for 60 min and then stained with SYBR gold. We excised a region of the gel corresponding to 300 to 500 bp and then recovered DNA using a QIAquick Gel Extraction kit. The ligated DNA was PCR amplified using adapter-specific primers and High-Fidelity DNA polymerase in 25 µL reaction volume for 12 cycles to minimize amplification. The concentrations of libraries were determined by qPCR using the Kapa SYBR qPCR Master mix kit and the PhiX library sample as a control. The final samples were diluted in 10 mM Tris-HCl, pH 8.0 and 0.05% Tween 20 and 2 nM of each DNA library was loaded onto a flow cell.

### Sequencing data generation and preprocessing

DNA libraries were sequenced using either single end (36 bp and 77 bp) or paired end (2×100 bp or 2×50 bp) protocols on a Illumina HiSeq 2000. Standard metrics were used to assess data quality. We used the *Saccharomyces cerevisiae* S288C reference genome, obtained from the SGD database on Feb 03, 2011 to align reads using BWA 0.5.9 [62]. We trimmed bases with base quality less than 20 from the 3′ end of each read. We removed reads with mapping quality less than 20. In addition, PCR duplicates were removed using Picard 1.57 (http://picard.sourceforge.net). We generated BAM files from all remaining reads using samtools 0.1.18 [63]. The average read depth of all sequenced strains is ∼160 X as shown in the Table S4.

### SNP and indel identification in clonal samples

To identify SNPs we used samtool 0.1.18 and bcftools 0.1.17 with the Bayesian inference option. We determined an empirical quality score cutoff of 160 using bcftools. For paired end sequencing data we excluded all anomalous read pairs. As clonal individuals are haploid we required SNP alleles to have call frequencies close to 1.0. In duplicated genomic regions or diploidized clones, which may contain heterozygous SNPs, we lowered this requirement to a call frequency near 0.5. In addition, we excluded all SNP calls that were also identified in the ancestral strain. To identify small insertions and deletions (indels) we used the DINDEL package [64]. We first generated candidate variants from BAM files using DINDEL, and then realigned each of them to the reference sequence in order to minimize false positive calls that are frequent in repetitive regions. Indels detected by DINDEL package are therefore defined as those that are shorter than the sequence read length (50 bp or 100 bp depending on sequencing mode).

### Identifying SNP alleles in heterogeneous population

We developed a heuristic threshold to identify low frequency SNPs in population sequencing data. First, we used two different BQ cutoffs, of 20 and 30, to identify SNPs using SNVer [65]. By comparing different population sequencing data to each other and to the ancestor, we identified SNPs in populations as ones that (1) are not found in the sequencing data from the ancestor and (2) exist uniquely in sequencing data from one population using both the high (30) and low (20) BQ cutoff options. We empirically found that optimal p-value cutoff of SNP calls generated using SNVer was 1×10^−8^, and the minimum total number of read counts covering the SNP location should be 50% of the average read counts in each population sequencing data. Using these heuristics we were able to detect SNPs with frequencies of at least 5% in population sequencing data. The allele frequency of each SNP in a population was determined by dividing the number of reads containing the alternative base by the total number of bases mapping to that position.

### Functional enrichment analysis

We collected all GO terms from ‘*GO.db*’ and ‘*org.Sc.sgd.db*’ packages in R, resulting in 6,366 ORFs assigned to 4,583 GO terms. We excluded any GO terms for which the number of assigned genes is less than 2 or more than 100. For a tested set of mutated genes we excluded ones without any GO annotation, incremented the count for each additional mutation identified in loci with multiple independent alleles and included both genes neighboring an intergenic SNPs. We then counted how many mutated loci are assigned to each term. We computed the p-value for each GO term using a one-tailed Fisher exact test. We used a Bonferroni correction to correct for multiple hypothesis testing.

### Drug sensitivity assays

We tested clones for sensitivity to 10 mM D-histidine (D-His) and 500 µM azetidine-2-carboxylate (ADCB), which are imported by nitrogen catabolite repression (NCR) regulated transporters [66]. We aimed to test drug sensitivities in both NCR-repressing and NCR-activating conditions. Therefore we used plates that containued either ammonium, which represses NCR-regulated genes or proline, which results in derepression of NCR-regulated genes [67]. Each mutant was first grown in liquid cultures containing YPD or SD plus ammonium sulfate (SD-AS). We then spotted normalized cell concentrations at ten-fold dilutions on solid agar containing SD-AS or SD plus 5 g/L proline (SD-Pro) with or without the drug. Sensitivity to drugs was determined following 2 days growth at 30°C.

### Estimation of allele and genotype dynamics

We prepared gDNA from population samples taken at 7 intermediate time point in addition to the final generation (i.e. 24, 61, 102, 137, 173, 213, and 250 generations) using a rapid gDNA extraction protocol [68]. We amplified 200–500 bp length amplicons that contain the SNP at a central position. All amplicons were sequenced using Sanger sequencing and the resulting electropherogram analyzed using PeakPicker to estimate allele frequencies as described [9], [69]. Vectors of allele frequencies were clustered and averaged if the Pearson correlation coefficient of two mutations was greater than 0.97 and the difference in allele frequencies in the final generation (based on deep sequencing) was less than 4%. As allele frequency estimates from Sanger sequencing are less accurate than those obtained from deep sequencing data we excluded a small number of allele frequency estimates derived from Sanger sequencing that were inconsistent with our deep sequencing results. All steps in this procedure are summarized in Figure S11.

### Measurement of genetic interactions among alleles

We backcrossed clone 3, recovered from the ammonium-limited condition to the ancestral strain of opposite mating type (FY5; MATα), sporulated the hybrid diploid and dissected tetrads. All segregants were tested for mating type using halo assays [70]. We obtained more than one hundred backcrossed strains bearing different combinations of the 5 mutations acquired by clone 3. Genomic DNA for each strain was prepared using a rapid DNA extraction protocol [68]. Genotyping was performed using allele specific PCR (the list of allele specific primers is presented in Table S5). Eight strains identified by this process contained all possible combinations of the three mutations of interest – *gat1-2*, *mep2-2* and *lst4-2* – and the ancestral alleles of the two additional loci (*RIM15* and *FAB1*) that were not studied. Each strain was individually competed against the mCitrine-labeled reference strains as described.

### Accession numbers

All DNA sequencing data are available from the NCBI Sequence Read Archive with accession number SRP032757. DNA microarray data are available through the NCBI Gene expression Omnibus with accession number GSE52787.

## Supporting Information

Figure S1 Batch culture screening of a random sample of 94 individuals from each adapted population. Mutants with distinct growth characteristics, as determined by growth rate and yield, were selected for further analysis.
(PDF)

Figure S2 Evidence of antagonistic pleiotropy in evolved lineages. Each mutant recovered from evolved populations was competed against a common fluorescently-labeled ancestral strain in batch cultures supplied with 5 g/L ammonium sulfate. Evolved clones exhibited fitness decreases of up to 4% in nitrogen-rich environments.
(PDF)

Figure S3 Complete aCGH results of all analyzed clones and populations that have undergone adaptive evolution in individual nitrogen sources. Most populations have acquired CNVs that include transporters of the specific nitrogen source except in the case of ammonium and arginine-limitation. For visualization, amplified or deleted regions with a minimum length of 10 kb and a log_2_ ratio >|0.5| are indicated by red (amplification) or green (deletion).
(PDF)

Figure S4 Identification of diploid and aneuploid cells. We performed flow cytometry analysis of DNA content of clones and compared them with haploid (FY4) and diploid (FY4/FY5) ancestral strains. Cytometry diagrams are 3D plots: different individuals lie along the y-axis, the z-axis is proportional to the DNA content and the x-axis indicates the per cell DNA content of the individual. Only the highest peak of each clone was compared to the reference strains' peaks in order to determine their ploidy. We also mated each clone to an isogenic MATα strain (FY5) and determined the viability of meiotic products, which is decreased in aneuploid lineages and extremely low for clones that had undergone a diploidization event.
(PDF)

Figure S5 Comparison of transcriptional divergence between clones using the distribution of pair-wise Pearson correlation coefficients as in [9]. Transcriptional divergence among clones adapted to nitrogen limitation is similar to that found for glucose- and phosphate-limited selections. Clones adapted to sulfur-limitation show far greater convergence of transcriptional states.
(PDF)

Figure S6 DNA copy number correlates with mRNA abundance. (A) CNVs result in increased gene expression. Nitrogen transporter genes located in CNVs tend to increase in expression with increased copy number. (B) All aneuploids identified showed increased mRNA expression of most genes in amplified chromosomes.
(PDF)

Figure S7 Allele frequencies distributions for each population based on whole genome sequencing. We estimated allele frequencies for all SNPs that were present at greater than ∼5% using deep sequencing read counts in 11 different nitrogen-limited populations.
(PDF)

Figure S8 CNVs are frequently selected in the presence of mixed nitrogen sources. Complete aCGH results for all populations and clones evolved in mixed nitrogen source environments. CNVs that include transporters for non-preferred nitrogen sources (urea, allantoin and proline) are preferentially selected when multiple nitrogen sources are present.
(PDF)

Figure S9 Significance analysis of NCR expression divergence in adapted clones. In most adaptations, NCR genes were significantly altered in expression. The statistical significance of NCR expression divergence (p-value) was calculated by 1) generating a null distribution by obtaining the mean absolute log_2_ gene expression ratio of 1,000 randomly chosen sets of 38 genes (without replacement) among all yeast ORFs on the microarray and then 2) computing the probability of obtaining an average absolute log2 gene expression ratio (indicated by a dotted red line) for the 38 measured NCR genes in the corresponding clone equal to or greater than that value. The greatest divergence in NCR expression is found among clones adapted to ammonium-limitation.
(PDF)

Figure S10 Drug sensitivity phenotypes of clonal isolates possessing *LST4* mutations. *Lst4* null mutants are resistant to the toxic proline analogue, azetidine-2-carboxylate (ADCB) [53] as it is required for proper trafficking of nitrogen permeases. Clones from the ammonium-limited population (c1 and c3) carrying mutations in *LST4* are not resistant to ADCB indicating that these are not loss of function mutations. As a control, adapted clones were also test for resistance to D-histidine, which is conferred by loss of function mutations in *GAP1*. Drug sensitivities were tested in both NCR derepressed (SD-P) and NCR repressed (SD-AS) conditions.
(PDF)

Figure S11 Procedure for estimating allele dynamics in the ammonium-limited population using Sanger sequencing. (A) Deep sequencing and Sanger sequencing showed good agreement as methods for inferring allele frequencies (R^2^ = 0.67441). (B) Allele frequency dynamics before clustering and normalization of alleles. (C) Clustering of all mutations based on the correlation in their allele frequency dynamics. We averaged the allele frequencies of mutations that fulfilled all of the following criteria: 1) occurred within the same clone, 2) had frequency dynamics with Pearson correlation coefficients >0.97 and 3) had differences in allele frequencies at the terminal generations less than 5% based on deep sequencing data. (D) Simplified allele dynamics model. Allele frequencies of less than 5% at earlier generations were excluded since they are below the level of background noise associated with estimation using Sanger sequencing.
(PDF)

Table S1 All mutations identified in clones.
(XLSX)

Table S2 All mutations identified in populations.
(XLSX)

Table S3 All mutations with minor frequency (∼5%) in the allantoin-limited adaptation, which contained a mutator phenotype.
(XLSX)

Table S4 Average sequence read depth of all sequenced populations and clones.
(PDF)

Table S5 List of primers used for allele specific PCR genotyping.
(PDF)

## References

[1] Dean AM, Thornton JW (2007) Mechanistic approaches to the study of evolution: the functional synthesis. Nature Reviews Genetics 8: 675–688 doi:10.1038/nrg2160 10.1038/nrg2160 PMC2488205 17703238

[2] Thomson JM, Gaucher EA, Burgan MF, De Kee DW, Li T, et al (2005) Resurrecting ancestral alcohol dehydrogenases from yeast. Nature genetics 37: 630–635 doi:10.1038/ng1553 15864308 10.1038/ng1553 PMC3618678

[3] Bridgham JT, Ortlund EA, Thornton JW (2009) An epistatic ratchet constrains the direction of glucocorticoid receptor evolution. Nature 461: 515–519 doi:10.1038/nature08249 19779450 10.1038/nature08249 PMC6141187

[4] Elena SF, Lenski RE (2003) Evolution experiments with microorganisms: the dynamics and genetic bases of adaptation. Nature Reviews Genetics 4: 457–469 doi:10.1038/nrg1088 10.1038/nrg1088 12776215

[5] Novick A, Szilard L (1950) Description of the chemostat. Science 112: 715–716. 14787503 10.1126/science.112.2920.715

[6] Monod J (1950) La technique de culture continue. Théorie et applications. Ann Inst Pasteur 79: 390–410.

[7] Lenski RE, Travisano M (1994) Dynamics of adaptation and diversification: a 10,000-generation experiment with bacterial populations. Proceedings of the National Academy of Sciences 91: 6808–6814. 10.1073/pnas.91.15.6808 PMC44287 8041701

[8] Lang GI, Botstein D, Desai MM (2011) Genetic variation and the fate of beneficial mutations in asexual populations. Genetics 188: 647–661 doi:10.1534/genetics.111.128942 21546542 10.1534/genetics.111.128942 PMC3176544

[9] Gresham D, Desai MM, Tucker CM, Jenq HT, Pai DA, et al (2008) The repertoire and dynamics of evolutionary adaptations to controlled nutrient-limited environments in yeast. PLoS Genet 4: e1000303 doi:10.1371/journal.pgen.1000303.t005 19079573 10.1371/journal.pgen.1000303 PMC2586090

[10] Gresham D, Usaite R, Germann SM, Lisby M, Botstein D, et al (2010) Adaptation to diverse nitrogen-limited environments by deletion or extrachromosomal element formation of the GAP1 locus. Proceedings of the National Academy of Sciences 107: 18551–18556 doi:10.1073/pnas.1014023107 10.1073/pnas.1014023107 PMC2972935 20937885

[11] Ferenci T (2007) Bacterial physiology, regulation and mutational adaptation in a chemostat environment. Advances in microbial physiology 53: 169–315 doi:10.1016/S0065-2911(07)53003-1 17707145 10.1016/S0065-2911(07)53003-1

[12] Kao KC, Sherlock G (2008) Molecular characterization of clonal interference during adaptive evolution in asexual populations of Saccharomyces cerevisiae. Nature genetics 40: 1499–1504 doi:10.1038/ng.280 19029899 10.1038/ng.280 PMC2596280

[13] Kvitek DJ, Sherlock G (2011) Reciprocal sign epistasis between frequently experimentally evolved adaptive mutations causes a rugged fitness landscape. PLoS Genet 7: e1002056 doi:10.1371/journal.pgen.1002056 21552329 10.1371/journal.pgen.1002056 PMC3084205

[14] Wenger JW, Piotrowski J, Nagarajan S, Chiotti K, Sherlock G, et al (2011) Hunger artists: yeast adapted to carbon limitation show trade-offs under carbon sufficiency. PLoS Genet 7: e1002202 doi:10.1371/journal.pgen.1002202 21829391 10.1371/journal.pgen.1002202 PMC3150441

[15] Magasanik B, Kaiser CA (2002) Nitrogen regulation in Saccharomyces cerevisiae. Gene 290: 1–18 doi:10.1016/S0378-1119(02)00558-9 12062797 10.1016/s0378-1119(02)00558-9

[16] Cooper TG (2002) Transmitting the signal of excess nitrogen in Saccharomyces cerevisiae from the Tor proteins to the GATA factors: connecting the dots. FEMS Microbiology Reviews 26: 223–238 doi:10.1111/j.1574-6976.2002.tb00612.x 12165425 10.1111/j.1574-6976.2002.tb00612.x PMC4384438

[17] Brown CJ, Todd KM, Rosenzweig RF (1998) Multiple duplications of yeast hexose transport genes in response to selection in a glucose-limited environment. Mol Biol Evol 15: 931–942. 9718721 10.1093/oxfordjournals.molbev.a026009

[18] Dunham MJ, Badrane H, Ferea T, Adams J, Brown PO, et al (2002) Characteristic genome rearrangements in experimental evolution of Saccharomyces cerevisiae. Proceedings of the National Academy of Sciences of the United States of America 99: 16144–16149 doi:10.1073/pnas.242624799 12446845 10.1073/pnas.242624799 PMC138579

[19] Zhong S, Khodursky A, Dykhuizen DE, Dean AM (2004) Evolutionary genomics of ecological specialization. Proceedings of the National Academy of Sciences of the United States of America 101: 11719 doi:10.1073/pnas.0404397101 15289609 10.1073/pnas.0404397101 PMC511043

[20] Zhang H, Zeidler AFB, Song W, Puccia CM, Malc E, et al (2013) Gene copy-number variation in haploid and diploid strains of the yeast Saccharomyces cerevisiae. Genetics 193: 785–801 doi:10.1534/genetics.112.146522 23307895 10.1534/genetics.112.146522 PMC3583998

[21] Dorsey M, Peterson C, Bray K, Paquin CE (1992) Spontaneous amplification of the ADH4 gene in Saccharomyces cerevisiae. Genetics 132: 943–950. 1459445 10.1093/genetics/132.4.943 PMC1205250

[22] Rancati G, Pavelka N, Fleharty B, Noll A, Trimble R, et al (2008) Aneuploidy underlies rapid adaptive evolution of yeast cells deprived of a conserved cytokinesis motor. Cell 135: 879–893 doi:10.1016/j.cell.2008.09.039 19041751 10.1016/j.cell.2008.09.039 PMC2776776

[23] Gerstein AC, Otto SP (2011) Cryptic fitness advantage: diploids invade haploid populations despite lacking any apparent advantage as measured by standard fitness assays. PloS one 6: e26599 doi:10.1371/journal.pone.0026599 22174734 10.1371/journal.pone.0026599 PMC3235103

[24] Torres EM, Sokolsky T, Tucker CM, Chan LY, Boselli M, et al (2007) Effects of aneuploidy on cellular physiology and cell division in haploid yeast. Science 317: 916–924 doi:10.1126/science.1142210 17702937 10.1126/science.1142210

[25] Lynch M, Sung W, Morris K, Coffey N, Landry CR, et al (2008) A genome-wide view of the spectrum of spontaneous mutations in yeast. Proceedings of the National Academy of Sciences of the United States of America 105: 9272–9277 doi:10.1073/pnas.0803466105 18583475 10.1073/pnas.0803466105 PMC2453693

[26] Araya CL, Payen C, Dunham MJ, Fields S (2010) Whole-genome sequencing of a laboratory-evolved yeast strain. BMC Genomics 11: 88 doi:10.1186/1471-2164-11-88 20128923 10.1186/1471-2164-11-88 PMC2829512

[27] Dettman JR, Rodrigue N, Melnyk AH, Wong A, Bailey SF, et al (2012) Evolutionary insight from whole-genome sequencing of experimentally evolved microbes. Mol Ecol 21: 2058–2077 doi:10.1111/j.1365-294X.2012.05484.x 22332770 10.1111/j.1365-294X.2012.05484.x

[28] Notley-McRobb L, Seeto S, Ferenci T (2003) The influence of cellular physiology on the initiation of mutational pathways in Escherichia coli populations. Proc Biol Sci 270: 843–848 doi:10.1098/rspb.2002.2295 12737663 10.1098/rspb.2002.2295 PMC1691312

[29] Herron MD, Doebeli M (2013) Parallel Evolutionary Dynamics of Adaptive Diversification in Escherichia coli. PLoS Biol 11: e1001490 doi:10.1371/journal.pbio.1001490 23431270 10.1371/journal.pbio.1001490 PMC3576414

[30] Maharjan R, Seeto S, Notley-McRobb L, Ferenci T (2006) Clonal adaptive radiation in a constant environment. Science 313: 514–517 doi:10.1126/science.1129865 16825532 10.1126/science.1129865

[31] Maharjan RP, Ferenci T, Reeves PR, Li Y, Liu B, et al (2012) The multiplicity of divergence mechanisms in a single evolving population. Genome Biology 13: R41 doi:10.1186/gb-2012-13-6-r41 22682524 10.1186/gb-2012-13-6-r41 PMC3446313

[32] Jin N, Chow CY, Liu L, Zolov SN, Bronson R, et al (2008) VAC14 nucleates a protein complex essential for the acute interconversion of PI3P and PI(3,5)P(2) in yeast and mouse. The EMBO Journal 27: 3221–3234 doi:10.1038/emboj.2008.248 19037259 10.1038/emboj.2008.248 PMC2600653

[33] Notley-McRobb L, King T, Ferenci T (2002) rpoS mutations and loss of general stress resistance in Escherichia coli populations as a consequence of conflict between competing stress responses. Journal of bacteriology 184: 806–811 doi:10.1128/JB.184.3.806-811.2002 11790751 10.1128/JB.184.3.806-811.2002 PMC139526

[34] Scherens B, Feller A, Vierendeels F, Messenguy F, Dubois E (2006) Identification of direct and indirect targets of the Gln3 and Gat1 activators by transcriptional profiling in response to nitrogen availability in the short and long term. FEMS Yeast Research 6: 777–791 doi:10.1111/j.1567-1364.2006.00060.x 16879428 10.1111/j.1567-1364.2006.00060.x

[35] Roberg KJ, Bickel S, Rowley N, Kaiser CA (1997) Control of amino acid permease sorting in the late secretory pathway of Saccharomyces cerevisiae by SEC13, LST4, LST7 and LST8. Genetics 147: 1569–1584. 9409822 10.1093/genetics/147.4.1569 PMC1208332

[36] Costanzo M, Baryshnikova A, Bellay J, Kim Y, Spear ED, et al (2010) The Genetic Landscape of a Cell. Science 327: 425–431 doi:10.1126/science.1180823 20093466 10.1126/science.1180823 PMC5600254

[37] Paquin C, Adams J (1983) Frequency of fixation of adaptive mutations is higher in evolving diploid than haploid yeast populations. Nature 302: 495–500. 6339947 10.1038/302495a0

[38] Paquin CE, Adams J (1983) Relative fitness can decrease in evolving asexual populations of S. cerevisiae. Nature 306: 368–370. 16752492 10.1038/306368a0

[39] Ferea TL, Botstein D, Brown PO, Rosenzweig RF (1999) Systematic changes in gene expression patterns following adaptive evolution in yeast. Proceedings of the National Academy of Sciences 96: 9721–9726 doi:10.1073/pnas.96.17.9721 10.1073/pnas.96.17.9721 PMC22277 10449761

[40] Gresham D, Ruderfer DM, Pratt SC, Schacherer J, Dunham MJ, et al (2006) Genome-wide detection of polymorphisms at nucleotide resolution with a single DNA microarray. Science 311: 1932–1936 doi:10.1126/science.1123726 16527929 10.1126/science.1123726

[41] Kubitschek HE (1970) Introduction to research with continuous cultures. Prentice Hall 195.

[42] Rudge SA, Anderson DM, Emr SD (2004) Vacuole size control: regulation of PtdIns(3,5)P2 levels by the vacuole-associated Vac14-Fig4 complex, a PtdIns(3,5)P2-specific phosphatase. Molecular Biology of the Cell 15: 24–36 doi:10.1091/mbc.E03-05-0297 14528018 10.1091/mbc.E03-05-0297 PMC307524

[43] Dove SK, McEwen RK, Mayes A, Hughes DC, Beggs JD, et al (2002) Vac14 controls PtdIns (3, 5) P2 synthesis and Fab1-dependent protein trafficking to the multivesicular body. Curr Biol 12: 885–893 doi:10.1016/S0960-9822(02)00891-6 12062051 10.1016/s0960-9822(02)00891-6

[44] Weisman LS (2003) Yeast vacuole inheritance and dynamics. Annu Rev Genet 37: 435–460 doi:10.1146/annurev.genet.37.050203.103207 14616069 10.1146/annurev.genet.37.050203.103207

[45] Li SC, Kane PM (2009) The yeast lysosome-like vacuole: endpoint and crossroads. Biochim Biophys Acta 1793: 650–663 doi:10.1016/j.bbamcr.2008.08.003 18786576 10.1016/j.bbamcr.2008.08.003 PMC2906225

[46] Cardenas ME, Cutler NS, Lorenz MC, Di Como CJ, Heitman J (1999) The TOR signaling cascade regulates gene expression in response to nutrients. Genes Dev 13: 3271–3279 doi:10.1101/gad.13.24.3271 10617575 10.1101/gad.13.24.3271 PMC317202

[47] Tamanoi F (2011) Ras signaling in yeast. Genes Cancer 2: 210–215 doi:10.1177/1947601911407322 21779494 10.1177/1947601911407322 PMC3128628

[48] Cameroni E, Hulo N, Roosen J, Winderickx J, De Virgilio C (2004) The novel yeast PAS kinase Rim15 orchestrates G0-associated antioxidant defense mechanisms. Cell Cycle 3: 460–466 doi:10.4161/cc.3.4.791 15300954

[49] Swinnen E, Wanke V, Roosen J, Smets B, Dubouloz F, et al (2006) Rim15 and the crossroads of nutrient signalling pathways in Saccharomyces cerevisiae. Cell Div 1: 3 doi:10.1186/1747-1028-1-3 16759348 10.1186/1747-1028-1-3 PMC1479807

[50] Ferenci T (2003) What is driving the acquisition of mutS and rpoS polymorphisms in Escherichia coli? Trends Microbiol 11: 457–461 doi:10.1016/j.tim.2003.08.003 14557028 10.1016/j.tim.2003.08.003

[51] Wang L, Spira B, Zhou Z, Feng L, Maharjan RP, et al (2010) Divergence involving global regulatory gene mutations in an Escherichia coli population evolving under phosphate limitation. Genome Biol Evol 2: 478–487 doi:10.1093/gbe/evq035 20639316 10.1093/gbe/evq035 PMC2997555

[52] Dunn B, Paulish T, Stanbery A, Piotrowski J, Koniges G, et al (2013) Recurrent Rearrangement during Adaptive Evolution in an Interspecific Yeast Hybrid Suggests a Model for Rapid Introgression. PLoS Genet 9: e1003366 doi:10.1371/journal.pgen.1003366 23555283 10.1371/journal.pgen.1003366 PMC3605161

[53] Rubio-Texeira M, Kaiser CA (2006) Amino acids regulate retrieval of the yeast general amino acid permease from the vacuolar targeting pathway. Molecular Biology of the Cell 17: 3031–3050 doi:10.1091/mbc.E05-07-0669 16641373 10.1091/mbc.E05-07-0669 PMC1483039

[54] Lee M-C, Marx CJ (2013) Synchronous waves of failed soft sweeps in the laboratory: remarkably rampant clonal interference of alleles at a single locus. Genetics 193: 943–952 doi:10.1534/genetics.112.148502 23307898 10.1534/genetics.112.148502 PMC3584008

[55] Gerke J, Lorenz K, Cohen B (2009) Genetic interactions between transcription factors cause natural variation in yeast. Science 323: 498–501 doi:10.1126/science.1166426 19164747 10.1126/science.1166426 PMC4984536

[56] Steiner CC, Weber JN, Hoekstra HE (2007) Adaptive variation in beach mice produced by two interacting pigmentation genes. PLoS Biol 5: e219 doi:10.1371/journal.pbio.0050219.g001 17696646 10.1371/journal.pbio.0050219 PMC1945039

[57] Hittinger CT, Gonçalves P, Sampaio JP, Dover J, Johnston M, et al (2010) Remarkably ancient balanced polymorphisms in a multi-locus gene network. Nature 464: 54–58 doi:10.1038/nature08791 20164837 10.1038/nature08791 PMC2834422

[58] Gietz RD, Schiestl RH (2007) High-efficiency yeast transformation using the LiAc/SS carrier DNA/PEG method. Nature protocols 2: 31–34 doi:10.1038/nprot.2007.13 17401334 10.1038/nprot.2007.13

[59] Brauer MJ, Huttenhower C, Airoldi EM, Rosenstein R, Matese JC, et al (2008) Coordination of Growth Rate, Cell Cycle, Stress Response, and Metabolic Activity in Yeast. Molecular Biology of the Cell 19: 352–367 doi:10.1091/mbc.E07-08-0779 17959824 10.1091/mbc.E07-08-0779 PMC2174172

[60] Kahm M, Hasenbrink G, Lichtenberg-Fraté H, Ludwig J, Kschischo M (2010) grofit: fitting biological growth curves with R. Journal of Statistical Software 33: 1–21.20808728

[61] Venkatraman ES, Olshen AB (2007) A faster circular binary segmentation algorithm for the analysis of array CGH data. Bioinformatics 23: 657–663 doi:10.1093/bioinformatics/btl646 17234643 10.1093/bioinformatics/btl646

[62] Li H, Durbin R (2010) Fast and accurate long-read alignment with Burrows-Wheeler transform. Bioinformatics 26: 589–595 doi:10.1093/bioinformatics/btp698 20080505 10.1093/bioinformatics/btp698 PMC2828108

[63] Li H, Handsaker B, Wysoker A, Fennell T, Ruan J, et al (2009) The Sequence Alignment/Map format and SAMtools. Bioinformatics 25: 2078–2079 doi:10.1093/bioinformatics/btp352 19505943 10.1093/bioinformatics/btp352 PMC2723002

[64] Albers CA, Lunter G, MacArthur DG, McVean G, Ouwehand WH, et al (2011) Dindel: accurate indel calls from short-read data. Genome Res 21: 961–973 doi:10.1101/gr.112326.110 20980555 10.1101/gr.112326.110 PMC3106329

[65] Wei Z, Wang W, Hu P, Lyon GJ, Hakonarson H (2011) SNVer: a statistical tool for variant calling in analysis of pooled or individual next-generation sequencing data. Nucleic Acids Research 39: e132 doi:10.1093/nar/gkr599 21813454 10.1093/nar/gkr599 PMC3201884

[66] Andréasson C, Neve EPA, Ljungdahl PO (2004) Four permeases import proline and the toxic proline analogue azetidine-2-carboxylate into yeast. Yeast 21: 193–199 doi:10.1002/yea.1052 14968425 10.1002/yea.1052

[67] Schure EG, Riel NA, Verrips CT (2000) The role of ammonia metabolism in nitrogen catabolite repression in Saccharomyces cerevisiae. FEMS Microbiology Reviews 24: 67–83 doi:10.1111/j.1574-6976.2000.tb00533.x 10640599 10.1111/j.1574-6976.2000.tb00533.x

[68] Lõoke M, Kristjuhan K, Kristjuhan A (2011) Extraction of genomic DNA from yeasts for PCR-based applications. BioTechniques 50: 325–328 doi:10.2144/000113672 21548894 10.2144/000113672 PMC3182553

[69] Ge B, Gurd S, Gaudin T, Dore C, Lepage P, et al (2005) Survey of allelic expression using EST mining. Genome Res 15: 1584–1591 doi:10.1101/gr.4023805 16251468 10.1101/gr.4023805 PMC1310646

[70] Julius D, Blair L, Brake A, Sprague G, Thorner J (1983) Yeast alpha factor is processed from a larger precursor polypeptide: the essential role of a membrane-bound dipeptidyl aminopeptidase. Cell 32: 839–852. 6339075 10.1016/0092-8674(83)90070-3

[71] Godard P, Urrestarazu A, Vissers S, Kontos K, Bontempi G, et al (2007) Effect of 21 different nitrogen sources on global gene expression in the yeast Saccharomyces cerevisiae. Molecular and Cellular Biology 27: 3065–3086 doi:10.1128/MCB.01084-06 17308034 10.1128/MCB.01084-06 PMC1899933

[72] Arnold K, Kiefer F, Kopp J, Battey JND, Podvinec M, et al (2009) The Protein Model Portal. J Struct Funct Genomics 10: 1–8 doi:10.1007/s10969-008-9048-5 19037750 10.1007/s10969-008-9048-5 PMC2704613

[73] Drotschmann K (1999) Mutator phenotypes of yeast strains heterozygous for mutations in the MSH2 gene. Proceedings of the National Academy of Sciences 96: 2970–2975 doi:10.1073/pnas.96.6.2970 10.1073/pnas.96.6.2970 PMC15879 10077621

[74] Barrick JE, Lenski RE (2009) Genome-wide mutational diversity in an evolving population of Escherichia coli. Cold Spring Harb Symp Quant Biol 74: 119–129 doi:10.1101/sqb.2009.74.018 19776167 10.1101/sqb.2009.74.018 PMC2890043

