# Supplementary figures and images for: Molecular Specificity, Convergence and Constraint Shape Adaptive Evolution in Nutrient-Poor Environments
Source: PLoS Genet. 2014 Jan 9;10(1):e1004041. doi: 10.1371/journal.pgen.1004041 (PMC3886903; doi:10.1371/journal.pgen.1004041)

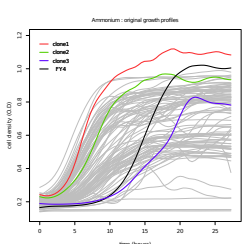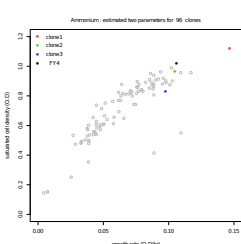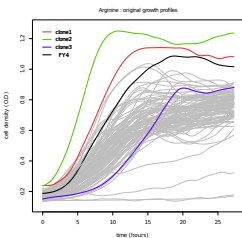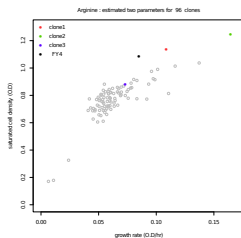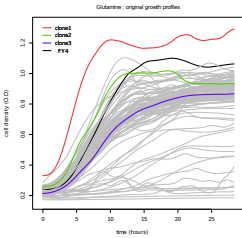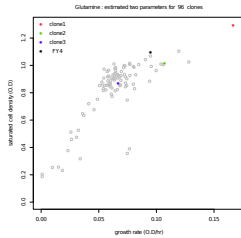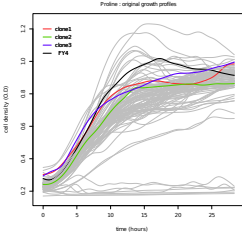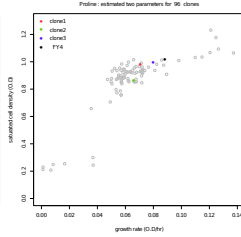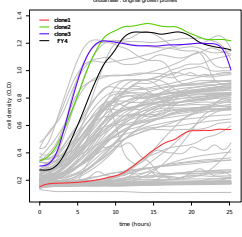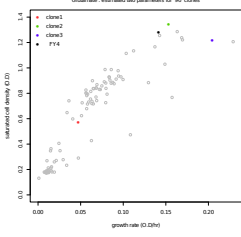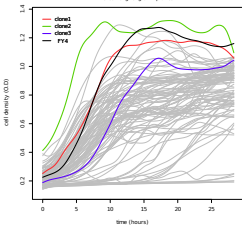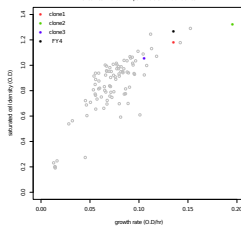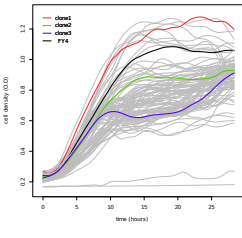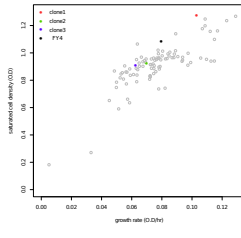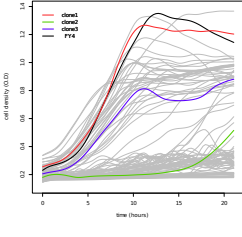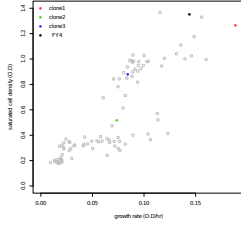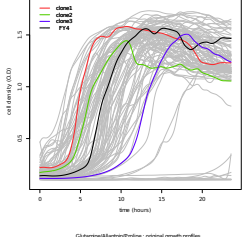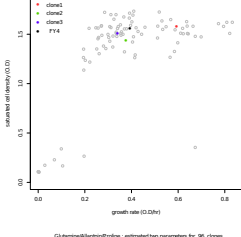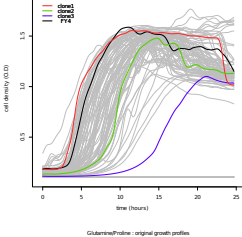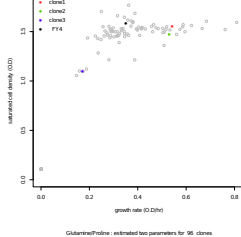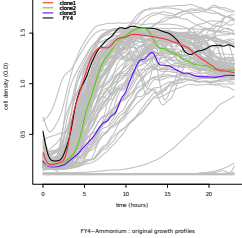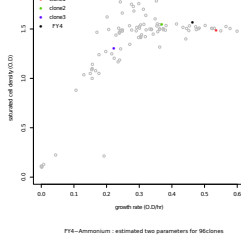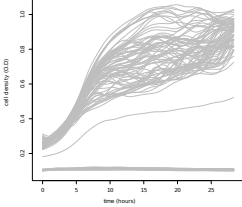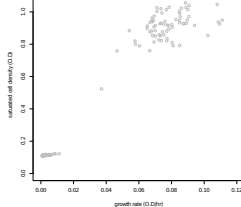

Supplement: Figure S1 — Batch culture screening of a random sample of 94 individuals from each adapted population. Mutants with distinct growth characteristics, as determined by growth rate and yield, were selected for further analysis. (PDF) [file pgen.1004041.s001.pdf]

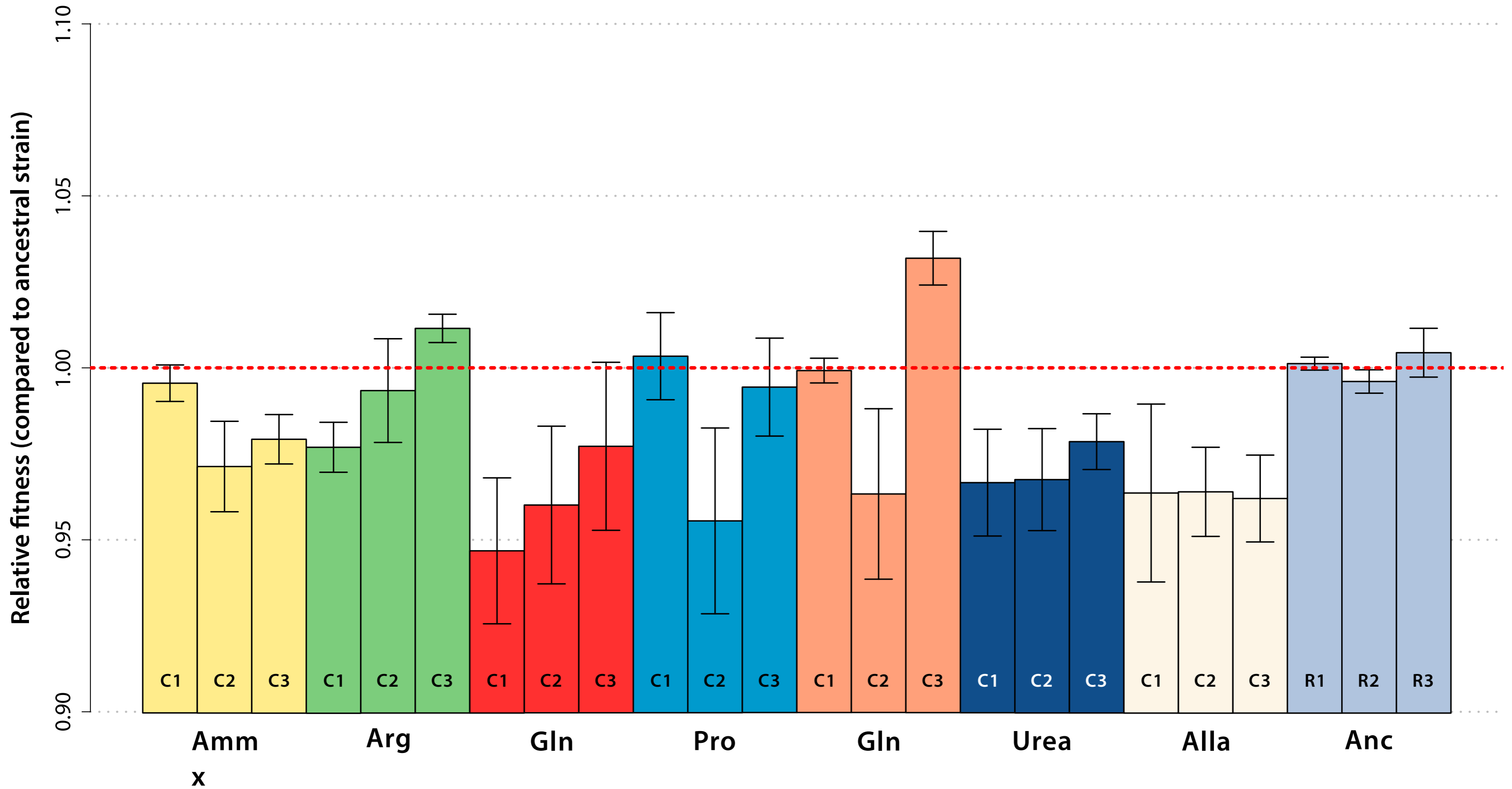

Supplement: Figure S2 — Evidence of antagonistic pleiotropy in evolved lineages. Each mutant recovered from evolved populations was competed against a common fluorescently-labeled ancestral strain in batch cultures supplied with 5 g/L ammonium sulfate. Evolved clones exhibited fitness decreases of up to 4% in nitrogen-rich environments. (PDF) [file pgen.1004041.s002.pdf]

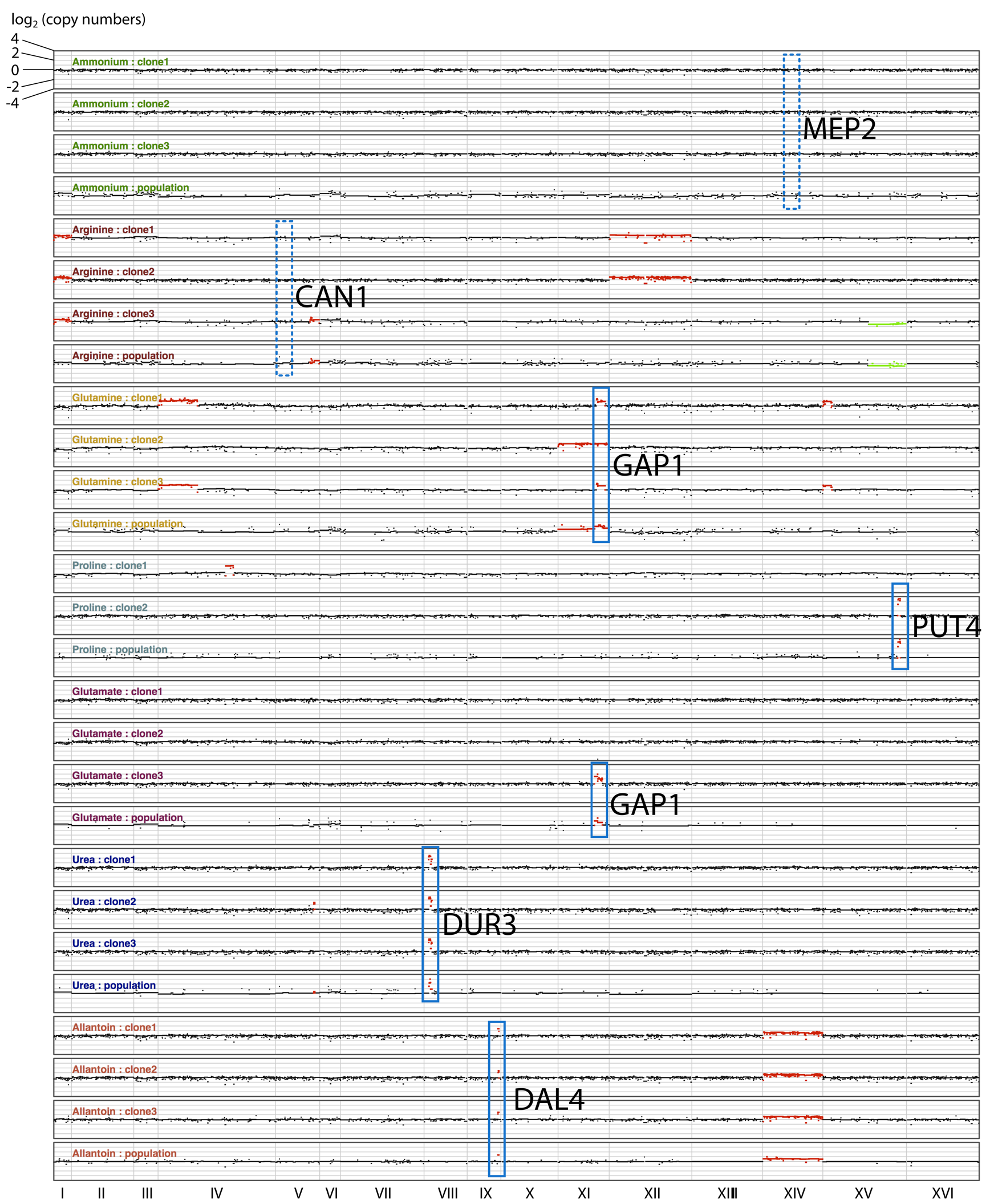

Supplement: Figure S3 — Complete aCGH results of all analyzed clones and populations that have undergone adaptive evolution in individual nitrogen sources. Most populations have acquired CNVs that include transporters of the specific nitrogen source except in the case of ammonium and arginine-limitation. For visualization, amplified or deleted regions with a minimum length of 10 kb and a log2 ratio >|0.5| are indicated by red (amplification) or green (deletion). (PDF) [file pgen.1004041.s003.pdf]

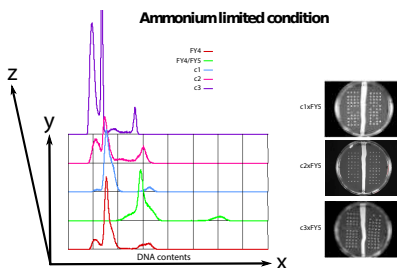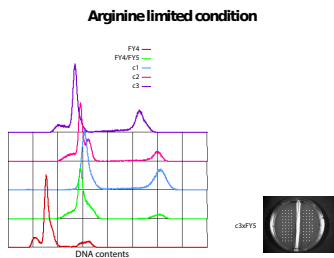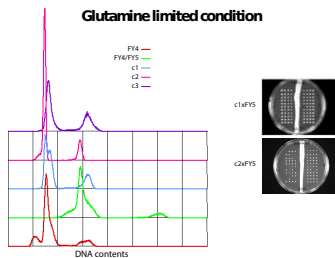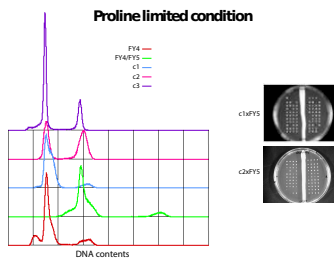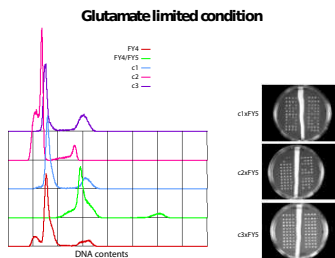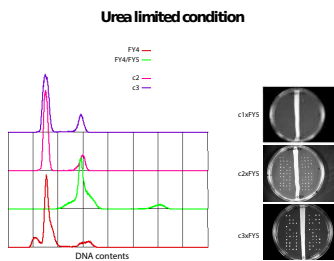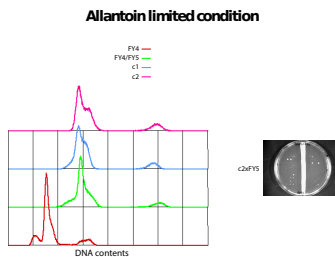

Supplement: Figure S4 — Identification of diploid and aneuploid cells. We performed flow cytometry analysis of DNA content of clones and compared them with haploid (FY4) and diploid (FY4/FY5) ancestral strains. Cytometry diagrams are 3D plots: different individuals lie along the y-axis, the z-axis is proportional to the DNA content and the x-axis indicates the per cell DNA content of the individual. Only the highest peak of each clone was compared to the reference strains' peaks in order to determine their ploidy. We also mated each clone to an isogenic MATα strain (FY5) and determined the viability of meiotic products, which is decreased in aneuploid lineages and extremely low for clones that had undergone a diploidization event. (PDF) [file pgen.1004041.s004.pdf]

# Estimated density of the distribution of PCC

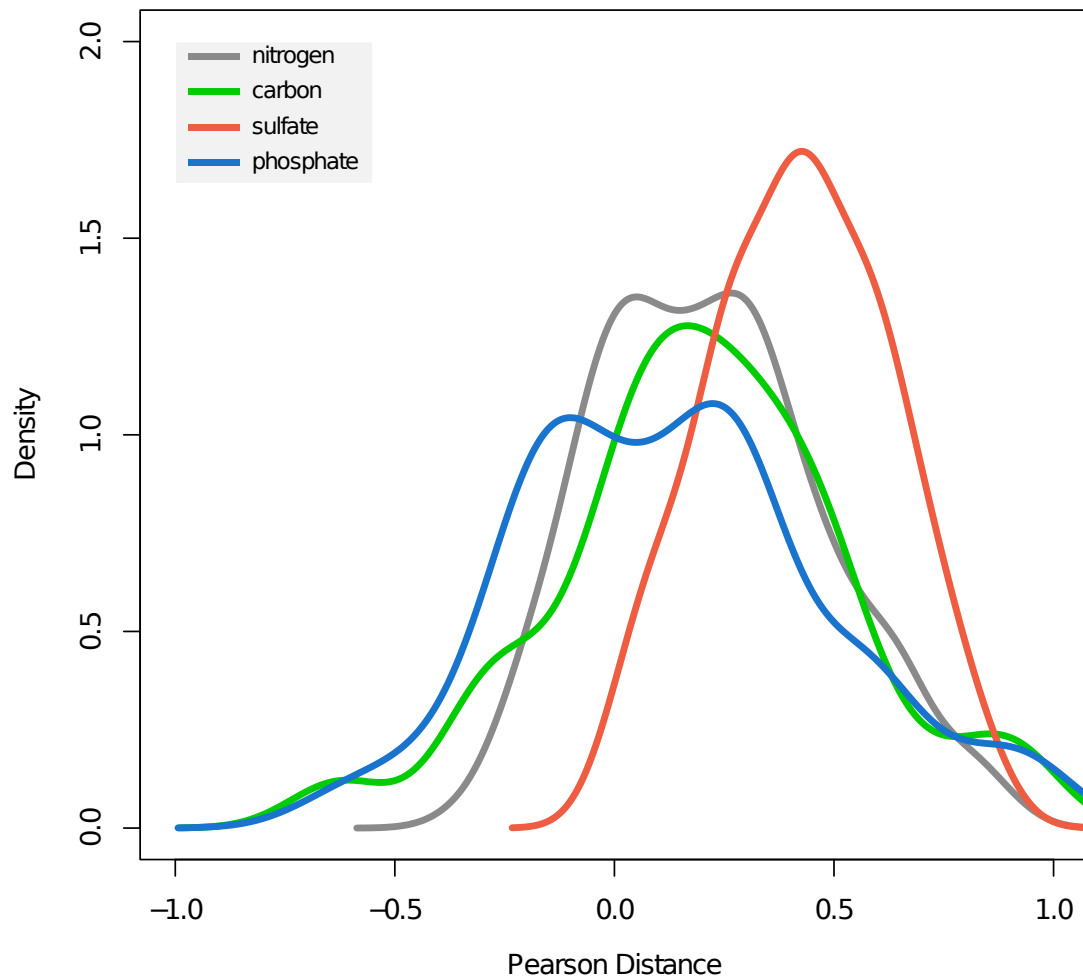

Supplement: Figure S5 — Comparison of transcriptional divergence between clones using the distribution of pair-wise Pearson correlation coefficients as in [9]. Transcriptional divergence among clones adapted to nitrogen limitation is similar to that found for glucose- and phosphate-limited selections. Clones adapted to sulfur-limitation show far greater convergence of transcriptional states. (PDF) [file pgen.1004041.s005.pdf]

**A**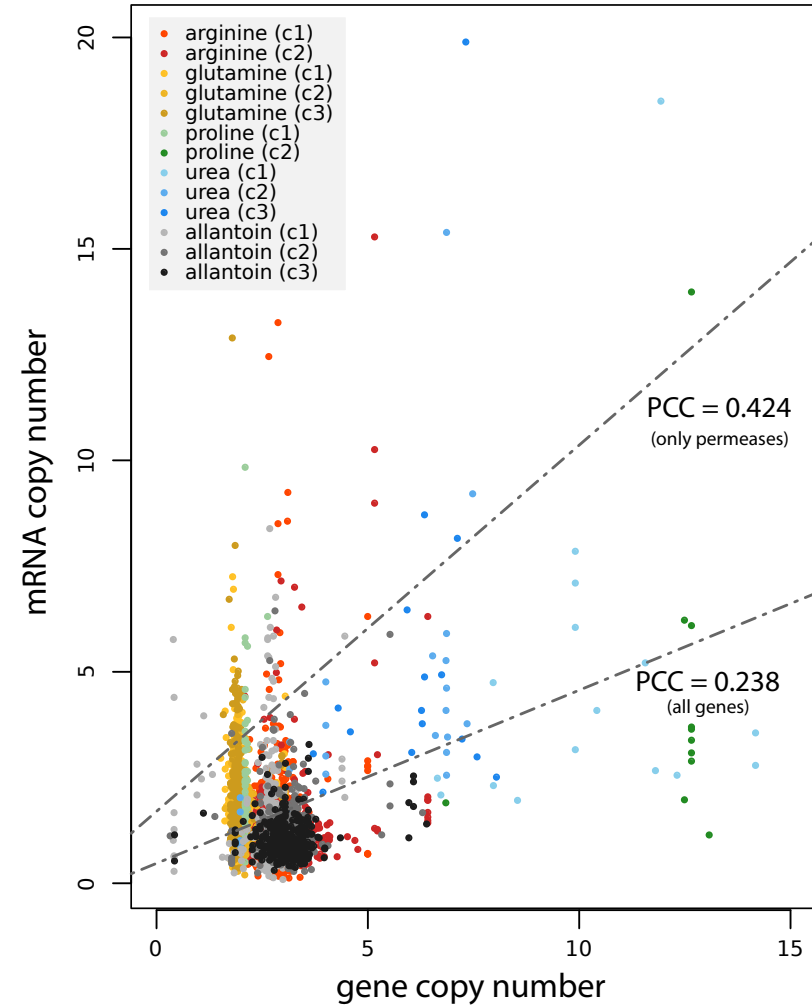**B**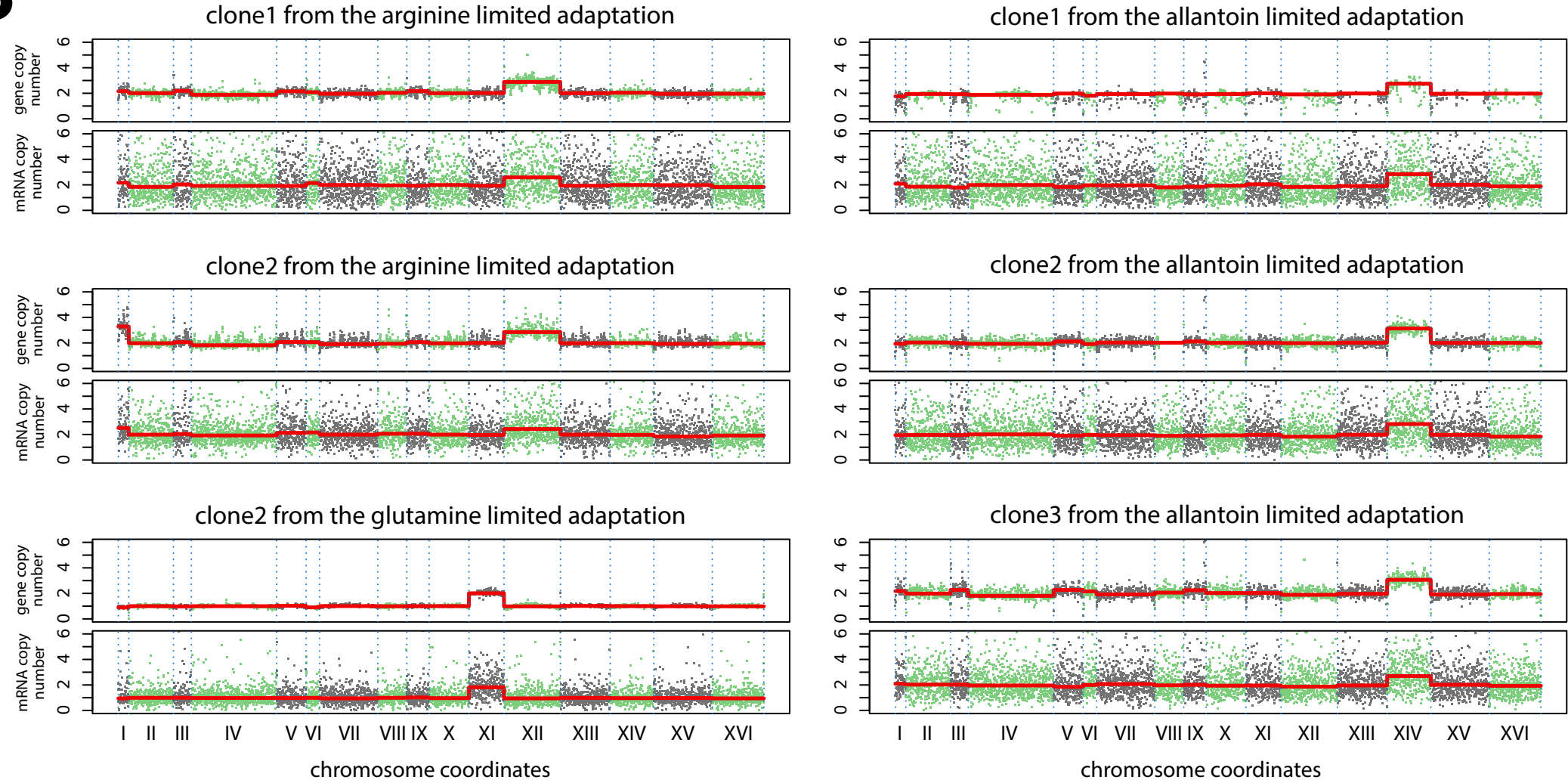

Supplement: Figure S6 — DNA copy number correlates with mRNA abundance. (A) CNVs result in increased gene expression. Nitrogen transporter genes located in CNVs tend to increase in expression with increased copy number. (B) All aneuploids identified showed increased mRNA expression of most genes in amplified chromosomes. (PDF) [file pgen.1004041.s006.pdf]

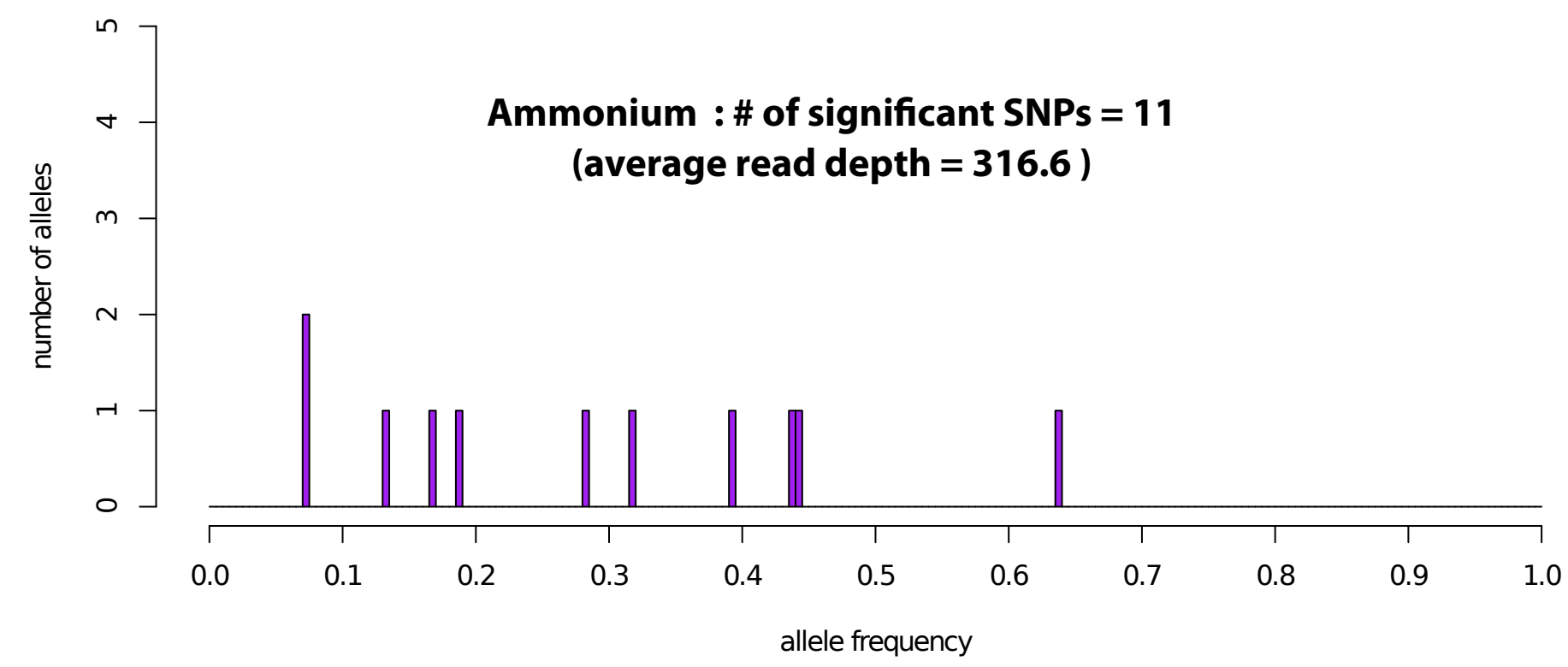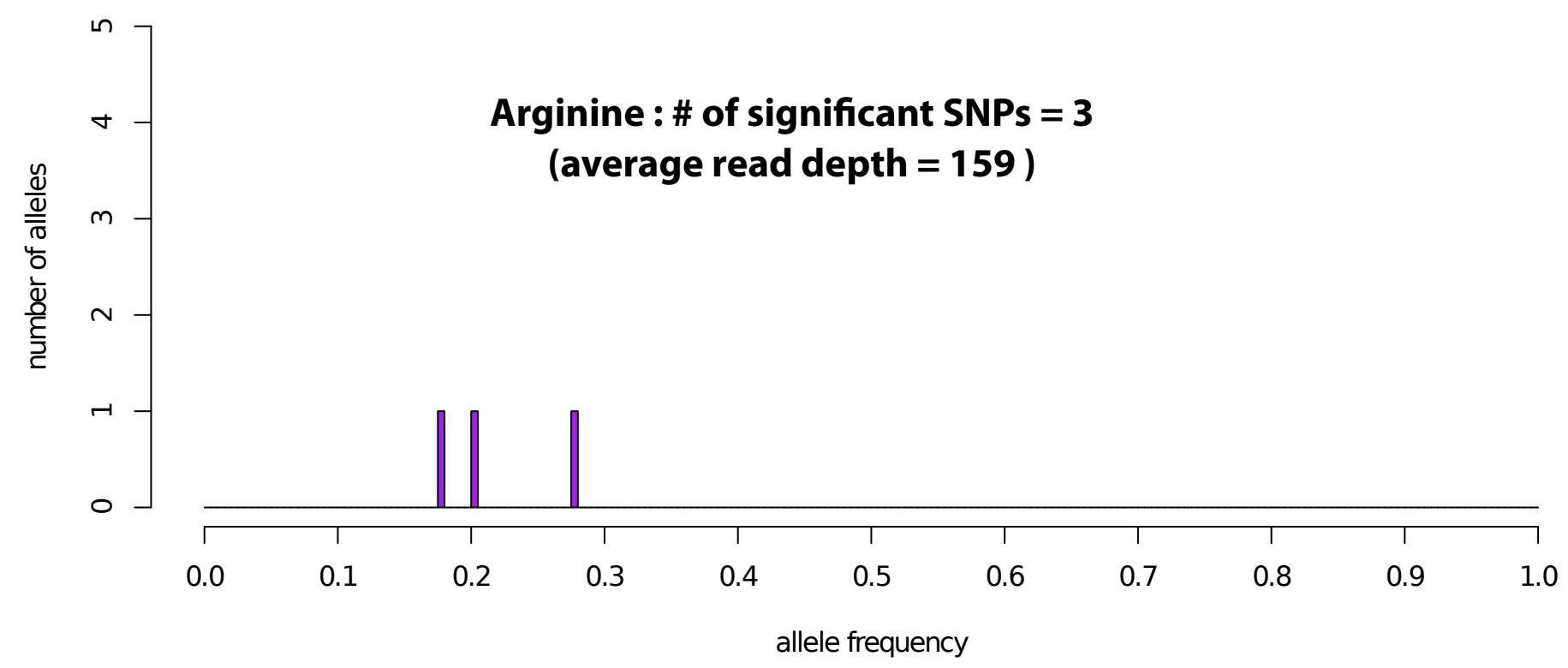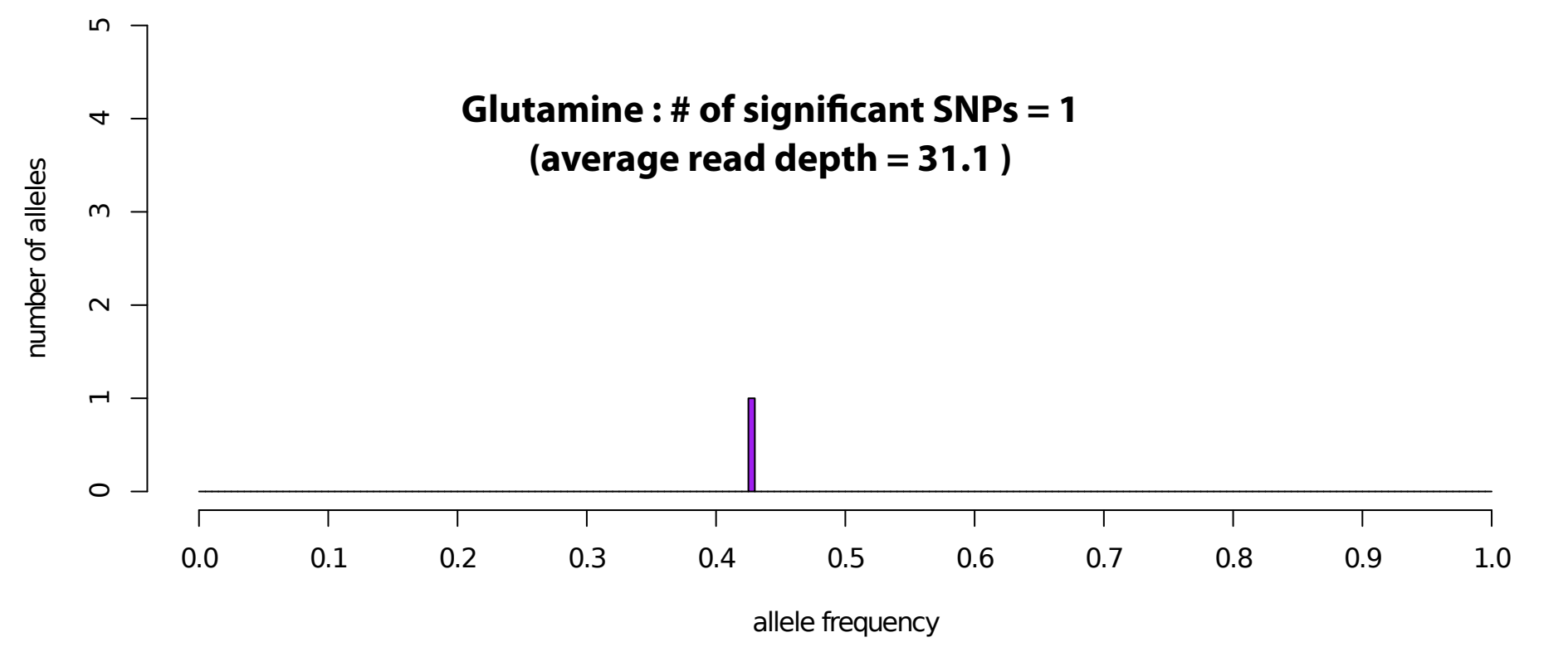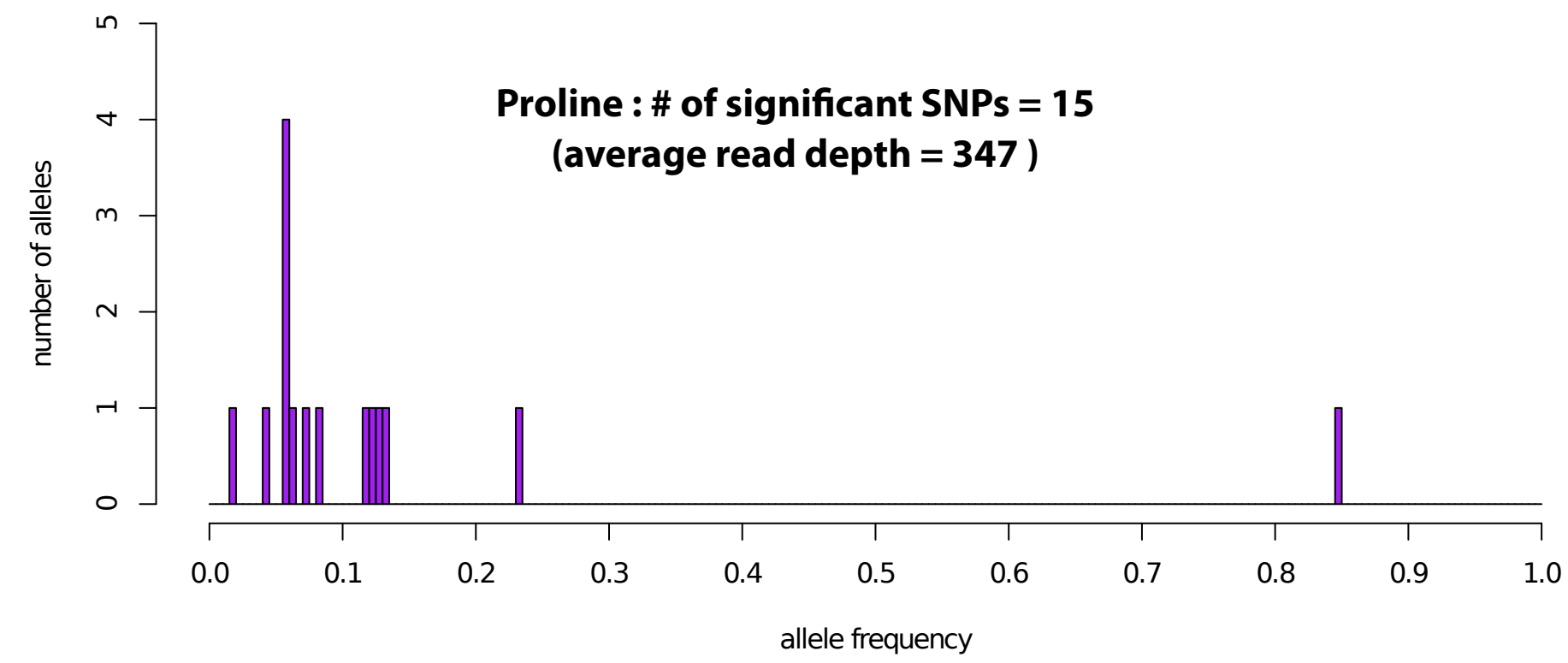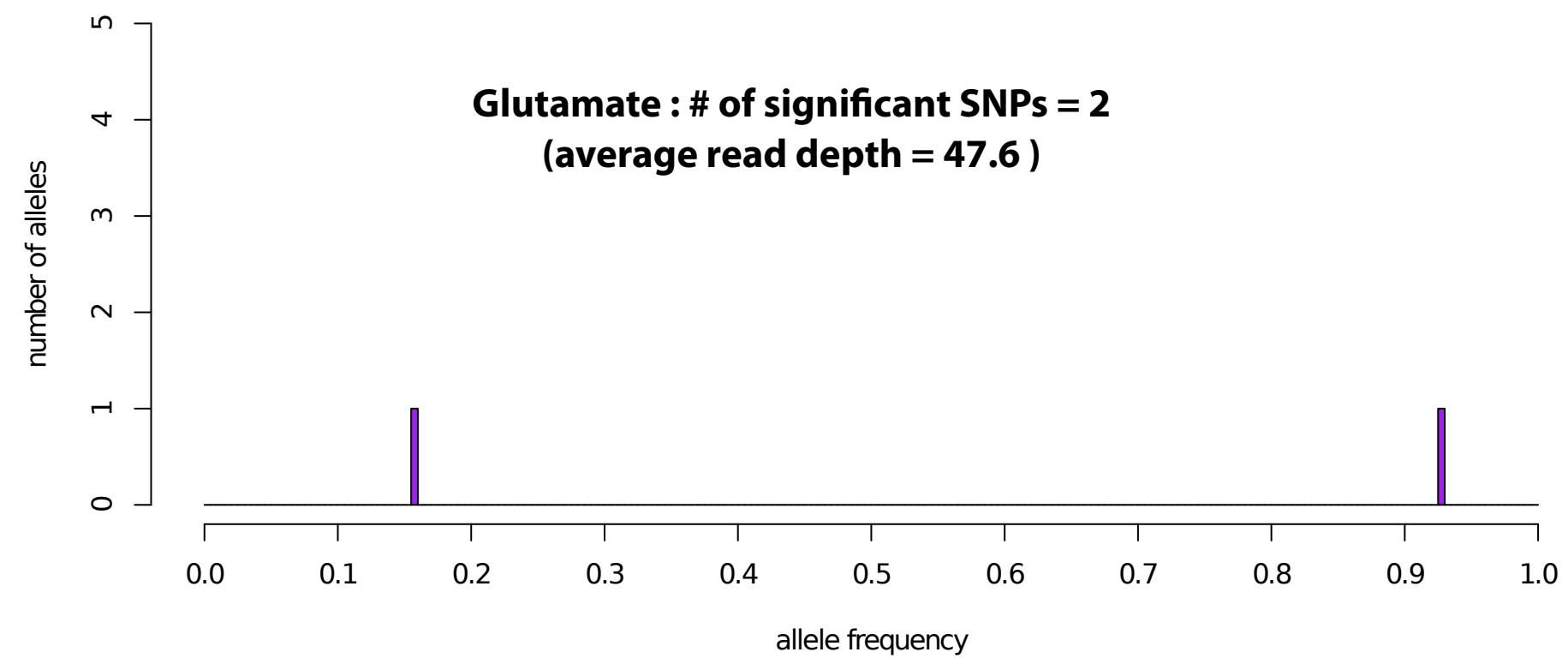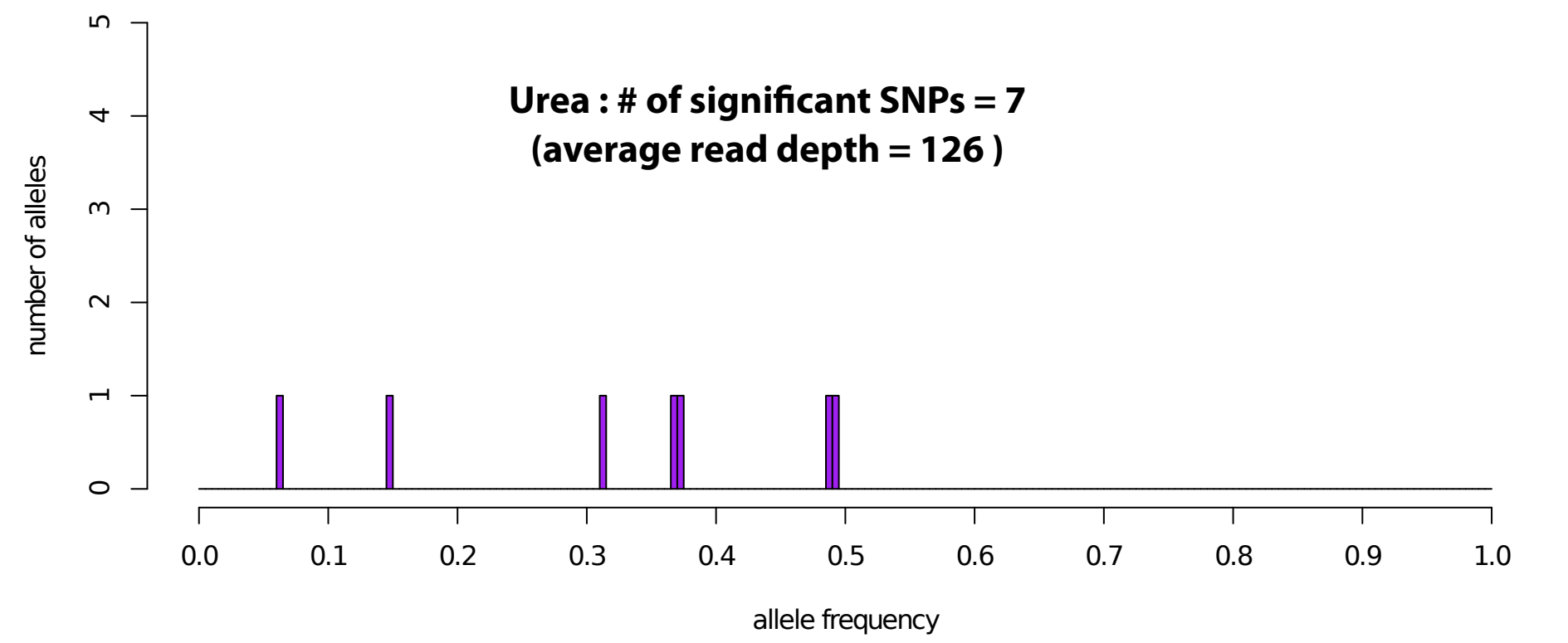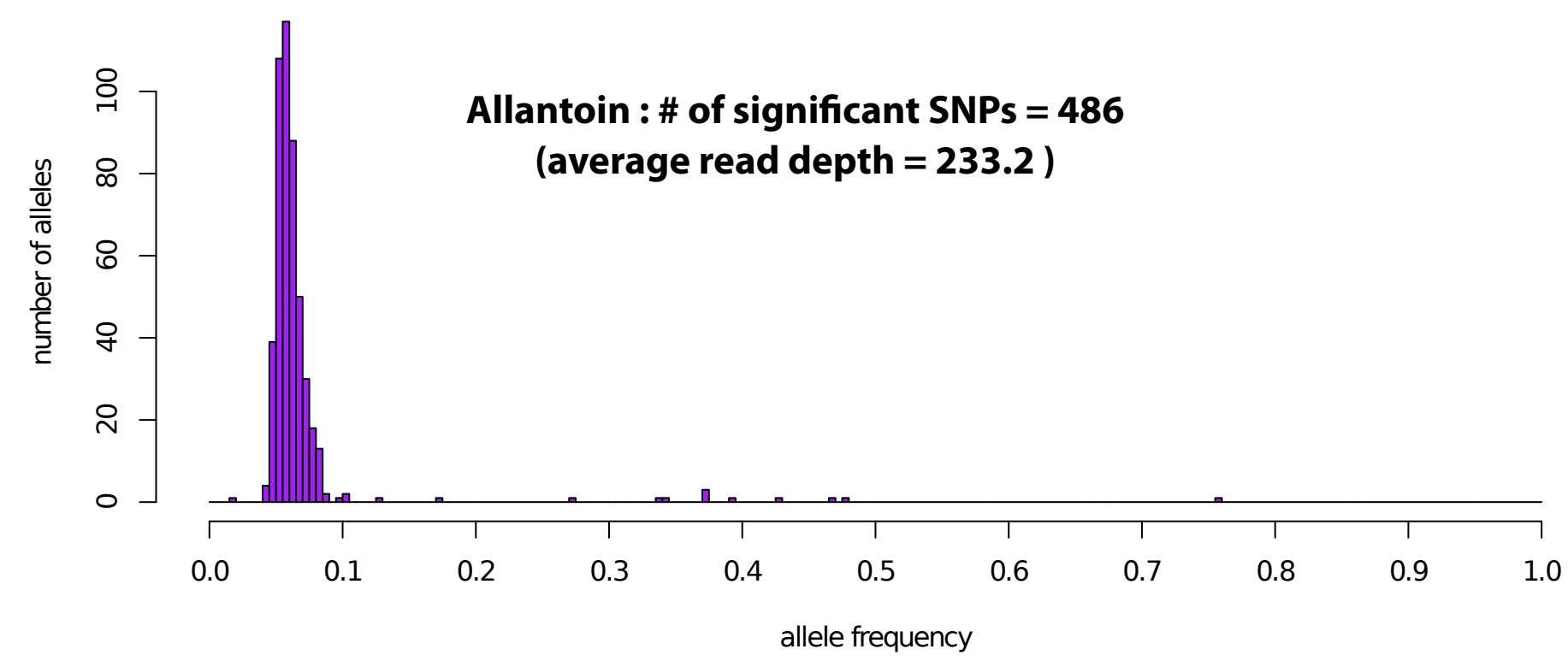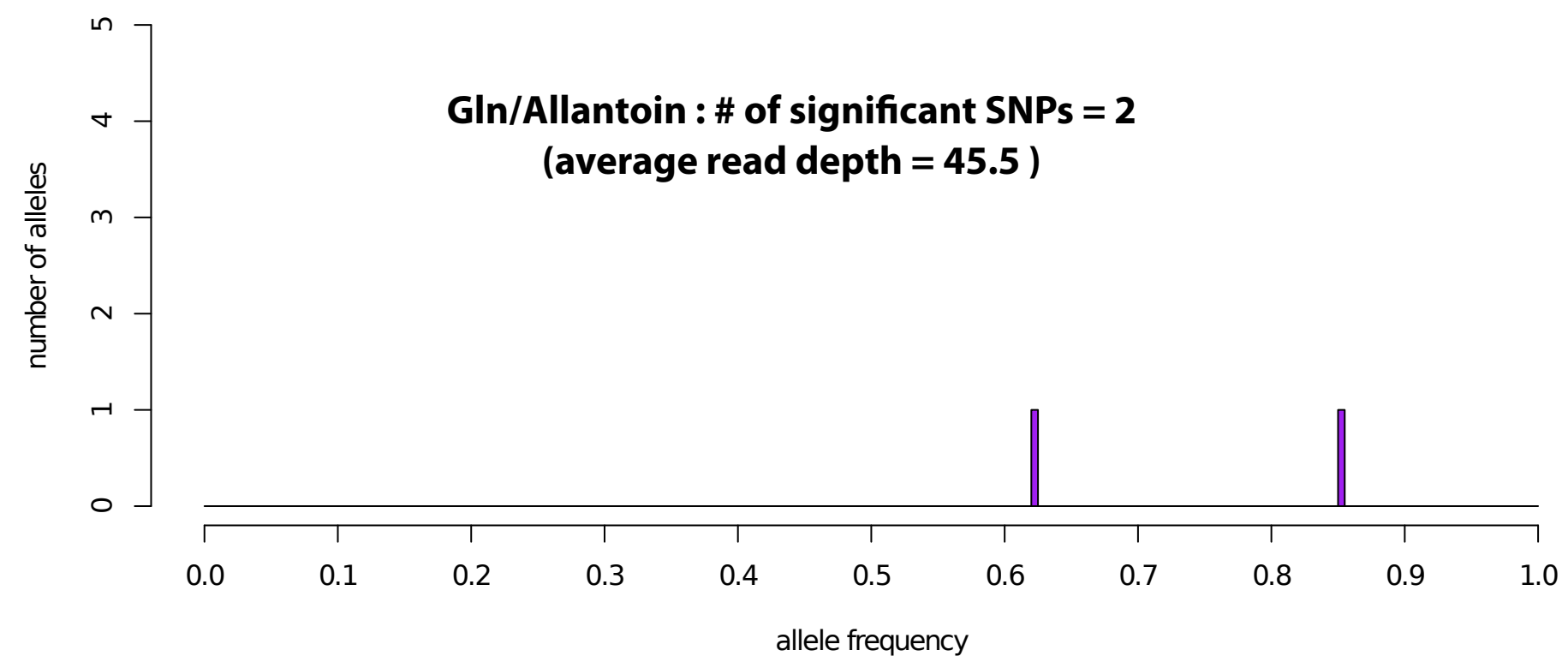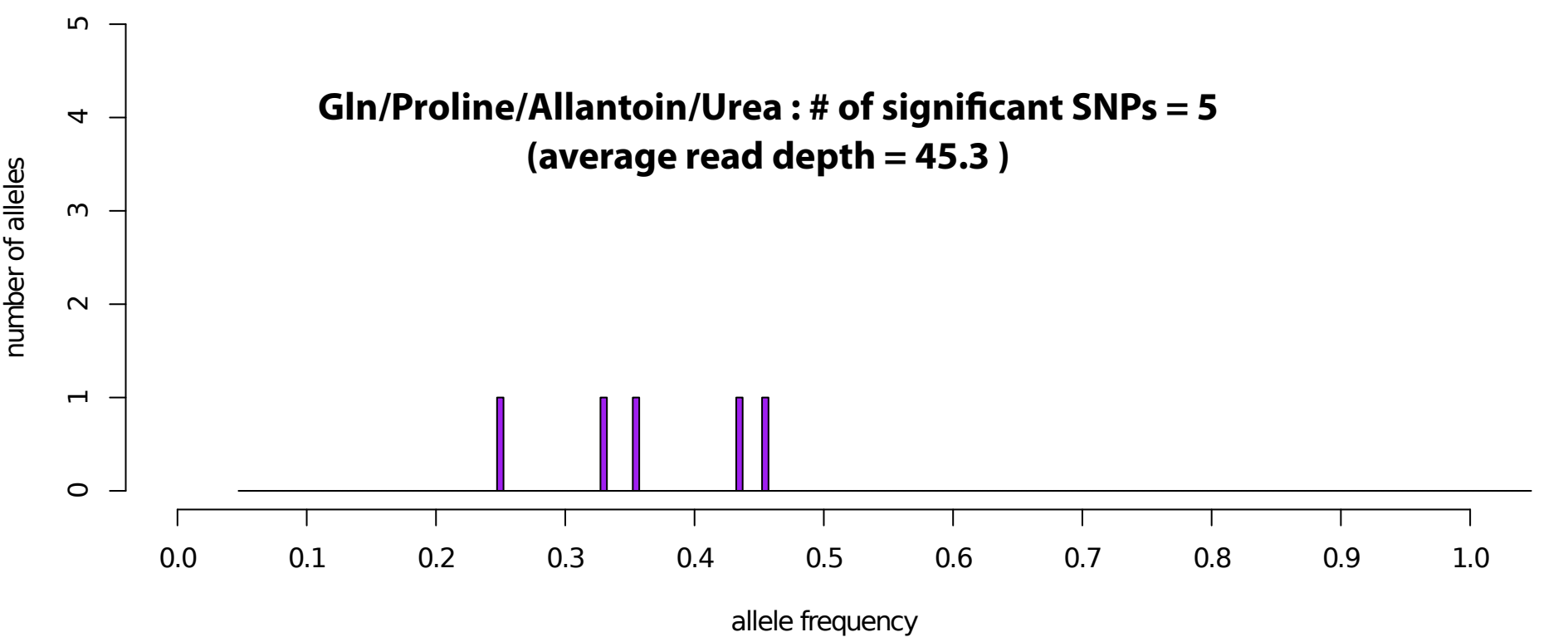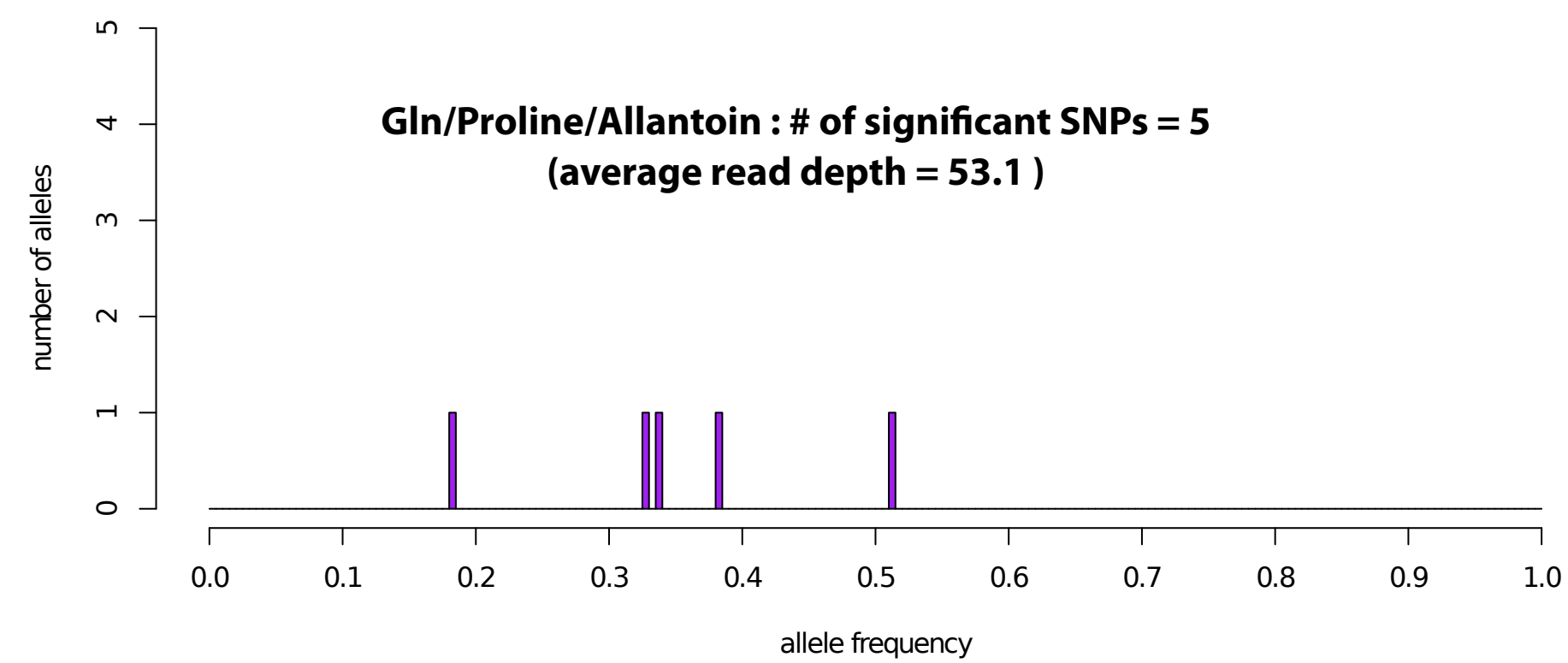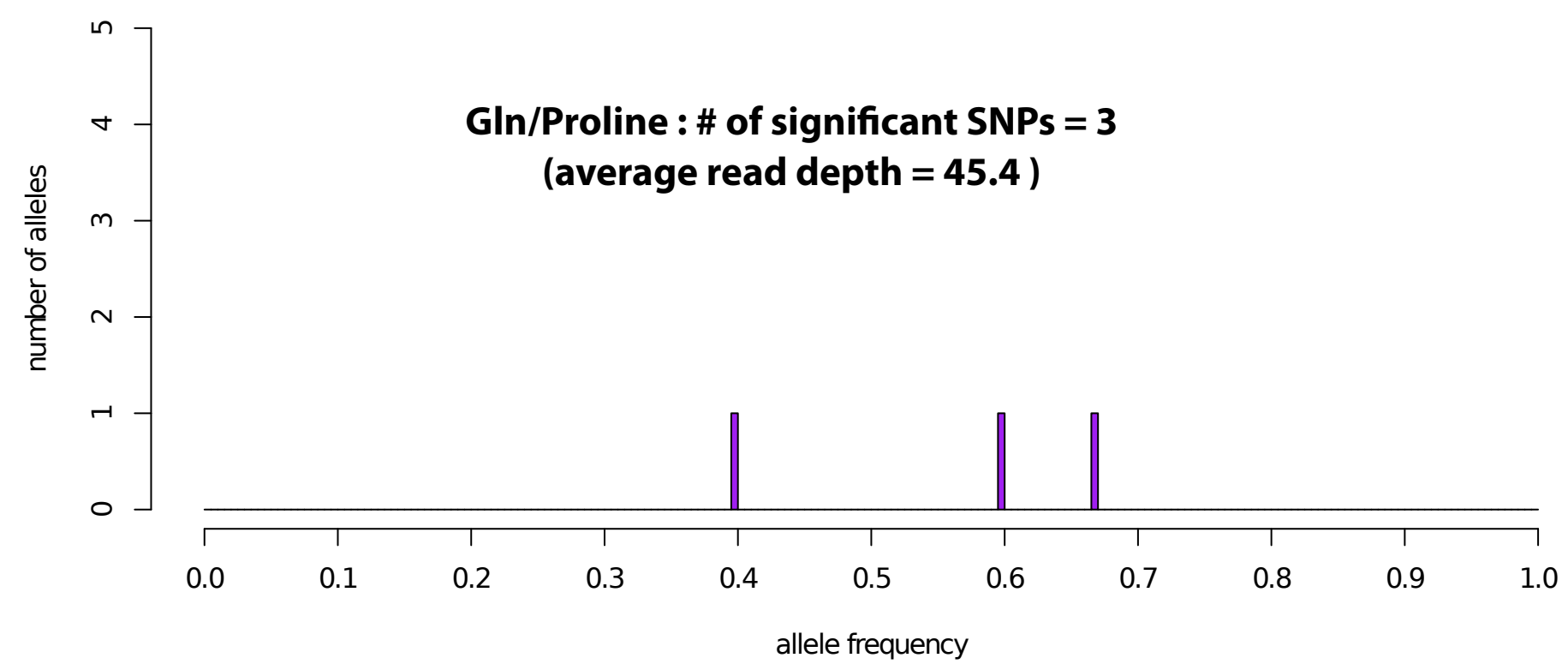

Supplement: Figure S7 — Allele frequencies distributions for each population based on whole genome sequencing. We estimated allele frequencies for all SNPs that were present at greater than ∼5% using deep sequencing read counts in 11 different nitrogen-limited populations. (PDF) [file pgen.1004041.s007.pdf]

$\log_2(\text{copy numbers})$

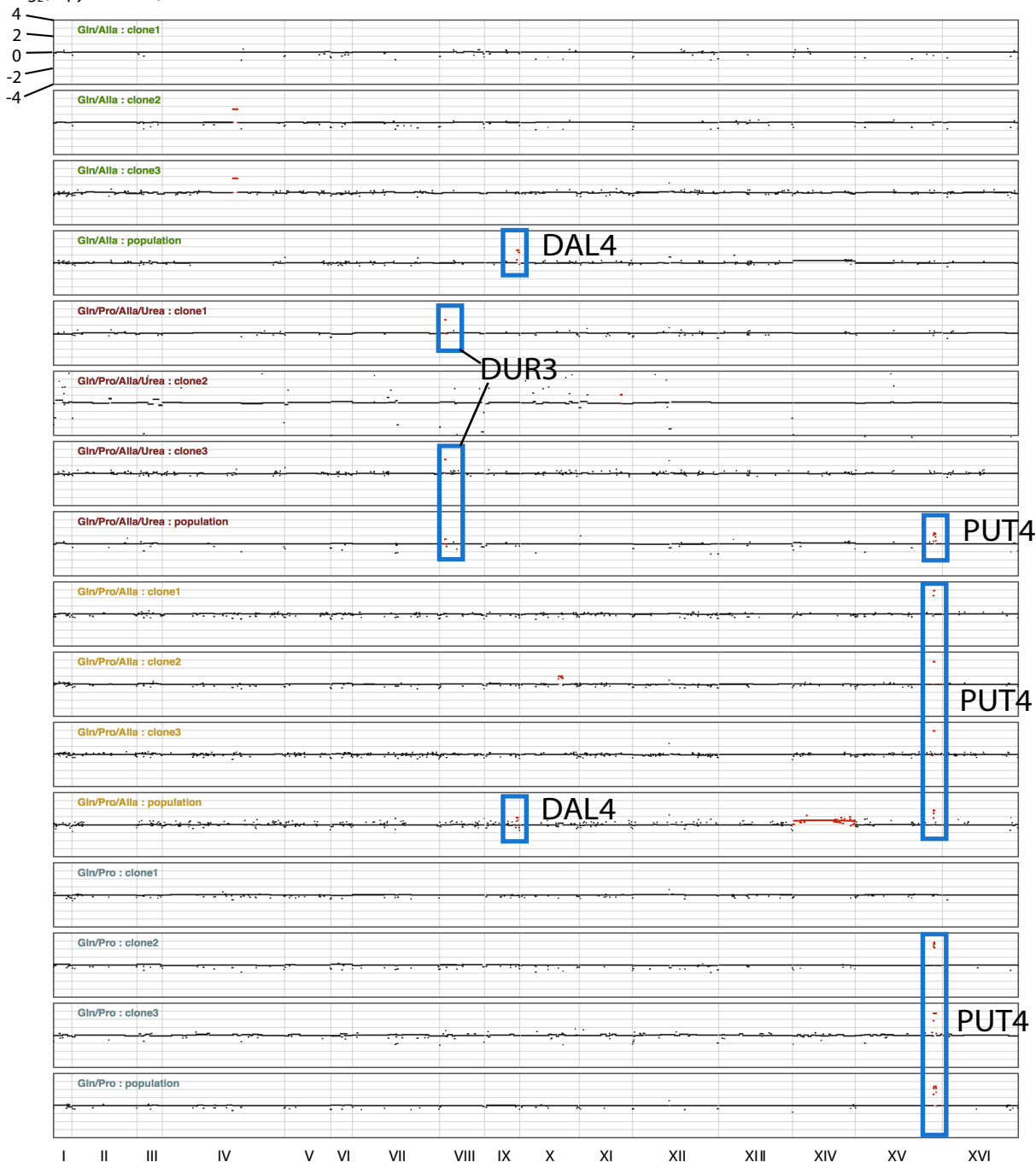

Supplement: Figure S8 — CNVs are frequently selected in the presence of mixed nitrogen sources. Complete aCGH results for all populations and clones evolved in mixed nitrogen source environments. CNVs that include transporters for non-preferred nitrogen sources (urea, allantoin and proline) are preferentially selected when multiple nitrogen sources are present. (PDF) [file pgen.1004041.s008.pdf]

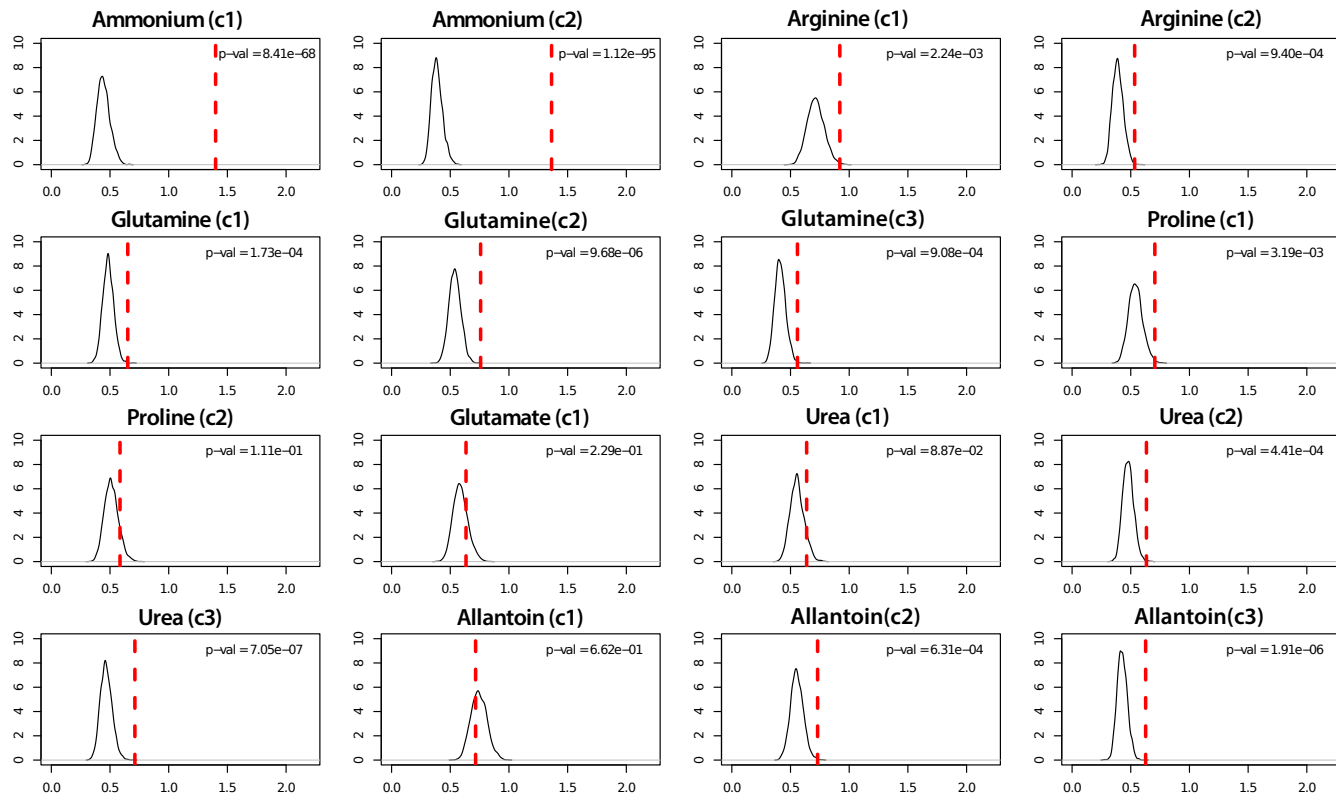

Supplement: Figure S9 — Significance analysis of NCR expression divergence in adapted clones. In most adaptations, NCR genes were significantly altered in expression. The statistical significance of NCR expression divergence (p-value) was calculated by 1) generating a null distribution by obtaining the mean absolute log2 gene expression ratio of 1,000 randomly chosen sets of 38 genes (without replacement) among all yeast ORFs on the microarray and then 2) computing the probability of obtaining an average absolute log2 gene expression ratio (indicated by a dotted red line) for the 38 measured NCR genes in the corresponding clone equal to or greater than that value. The greatest divergence in NCR expression is found among clones adapted to ammonium-limitation. (PDF) [file pgen.1004041.s009.pdf]

## 400 $\mu$ M Ammonium Sulfate evolution

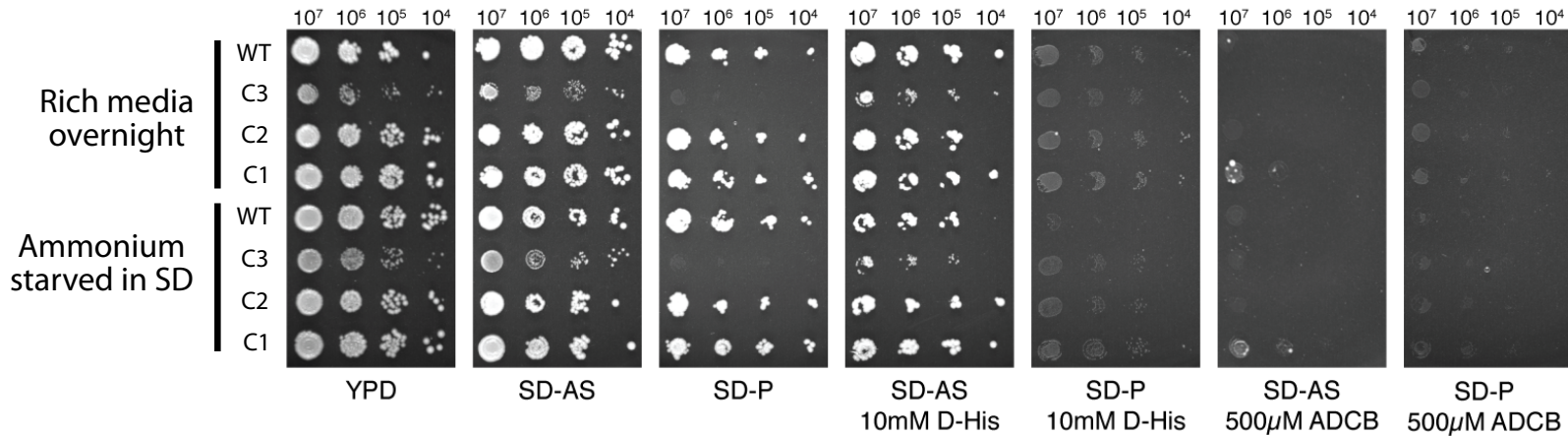

Supplement: Figure S10 — Drug sensitivity phenotypes of clonal isolates possessing LST4 mutations. Lst4 null mutants are resistant to the toxic proline analogue, azetidine-2-carboxylate (ADCB) [53] as it is required for proper trafficking of nitrogen permeases. Clones from the ammonium-limited population (c1 and c3) carrying mutations in LST4 are not resistant to ADCB indicating that these are not loss of function mutations. As a control, adapted clones were also test for resistance to D-histidine, which is conferred by loss of function mutations in GAP1. Drug sensitivities were tested in both NCR derepressed (SD-P) and NCR repressed (SD-AS) conditions. (PDF) [file pgen.1004041.s010.pdf]
