# Supplementary material for: Molecular Specificity, Convergence and Constraint Shape Adaptive Evolution in Nutrient-Poor Environments
Source: PLoS Genet. 2014 Jan 9;10(1):e1004041. doi: 10.1371/journal.pgen.1004041 (PMC3886903; doi:10.1371/journal.pgen.1004041)

A

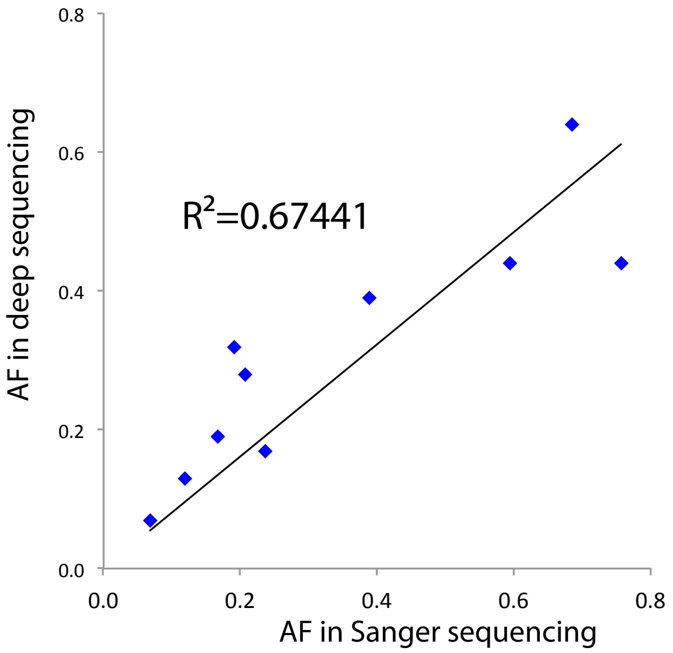

B

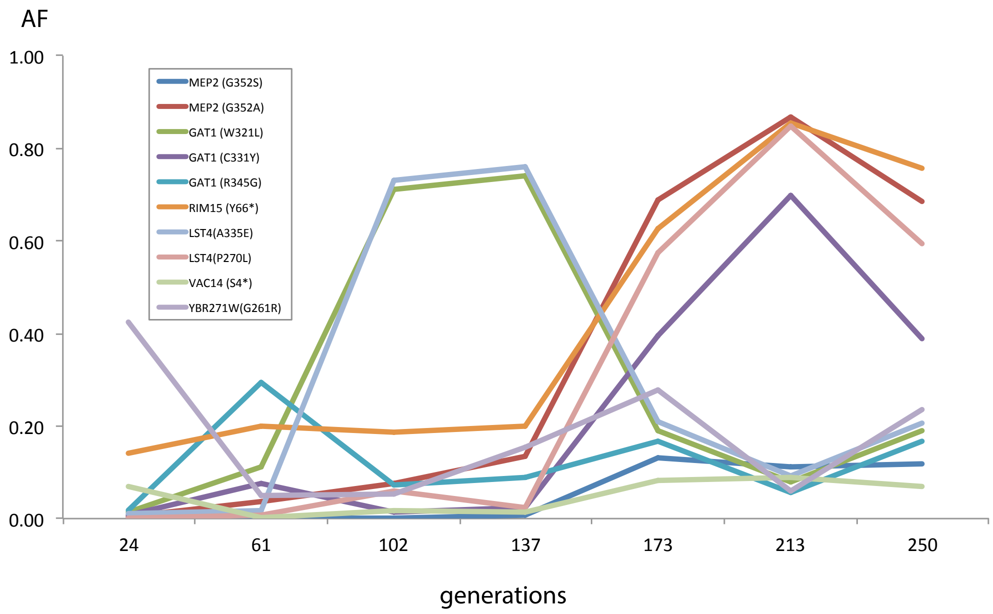

C

	AF *	MEP2 (G352S)	GAT1 (W321L)	LST4 (A335E)	GAT1 (R345G)	YBR271W (G261R)	VAC14 (S4*)	MEP2 (G352A)	GAT1 (C331Y)	RIM15 (Y66*)	LST4 (P270L)
MEP2 (G352S)	0.13	1.00	-0.38	-0.31	0.75	0.71	0.96	0.97	0.88	0.95	0.94
GAT1 (W321L)	0.32		1.00	**0.99**	0.08	0.07	-0.29	-0.35	-0.47	-0.40	-0.40
LST4 (A335E)	0.28	Clone1		1.00	0.13	0.12	-0.22	-0.28	-0.41	-0.33	-0.33
GAT1 (R345G)	0.19				1.00	**0.97**	0.64	0.61	0.39	0.58	0.53
YBR271W (G261R)	0.17					1.00	0.57	0.56	0.33	0.51	0.46
VAC14 (S4*)	0.07						1.00	0.99	0.94	0.97	0.98
MEP2 (G352A)	0.64				Clone2			1.00	0.96	0.99	0.99
GAT1 (C331Y)	0.39								1.00	**0.97**	**0.98**
RIM15 (Y66*)	0.44									1.00	**0.99**
LST4 (P270L)	0.44							Clone3			1.00

D

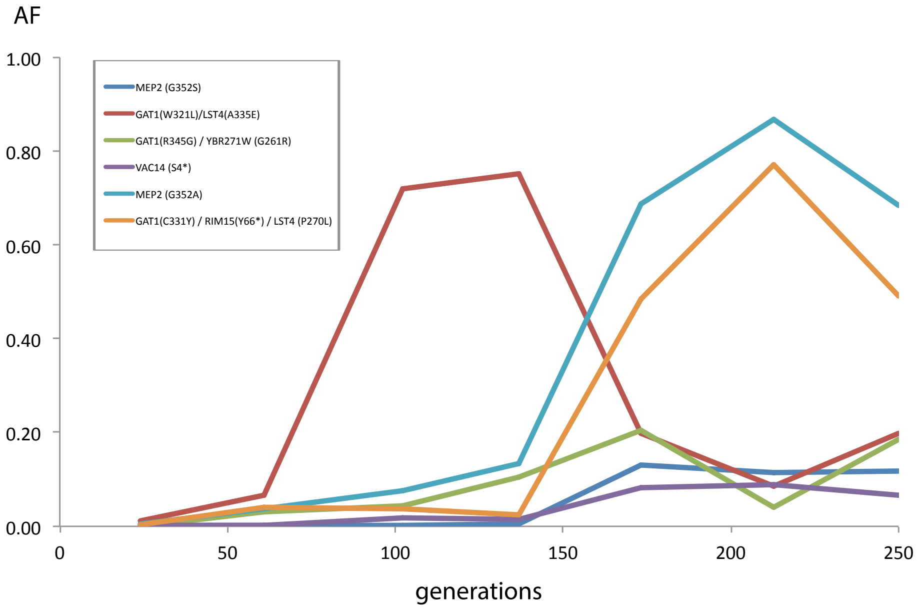

Supplement: Figure S11 — Procedure for estimating allele dynamics in the ammonium-limited population using Sanger sequencing. (A) Deep sequencing and Sanger sequencing showed good agreement as methods for inferring allele frequencies (R2 = 0.67441). (B) Allele frequency dynamics before clustering and normalization of alleles. (C) Clustering of all mutations based on the correlation in their allele frequency dynamics. We averaged the allele frequencies of mutations that fulfilled all of the following criteria: 1) occurred within the same clone, 2) had frequency dynamics with Pearson correlation coefficients >0.97 and 3) had differences in allele frequencies at the terminal generations less than 5% based on deep sequencing data. (D) Simplified allele dynamics model. Allele frequencies of less than 5% at earlier generations were excluded since they are below the level of background noise associated with estimation using Sanger sequencing. (PDF) [file pgen.1004041.s011.pdf]
