# Supplementary material for: Molecular Specificity, Convergence and Constraint Shape Adaptive Evolution in Nutrient-Poor Environments
Source: PLoS Genet. 2014 Jan 9;10(1):e1004041. doi: 10.1371/journal.pgen.1004041 (PMC3886903; doi:10.1371/journal.pgen.1004041)
Supplement: Table S4 — Average sequence read depth of all sequenced populations and clones. (PDF) [file pgen.1004041.s015.pdf]

Nitrogen source	sample	sequencing type	average RD
Ammonium	C1	100x100	316.8
Ammonium	C2	100x100	339.9
Ammonium	C3	100x100	271.7
Ammonium	population	100x100	316.6
Arginine	C1	100x100	99.9
Arginine	C2	100x100	134.1
Arginine	C3	100x100	142.7
Arginine	population	100x100	159.0
Glutamine	C1	100x100	259.6
Glutamine	C2	100x100	616.4
Glutamine	C3	100x100	443.5
Glutamine	population	77	31.1
Proline	C1	36	20.5
Proline	C2	100x100	144.7
Proline	population	100x100	347.0

Nitrogen source	sample	sequencing type	average RD
Glutamate	C1	50x50	14.9
Glutamate	population	77	47.6
Urea	C1	50x50	38.6
Urea	C2	50x50	38.3
Urea	C3	50x50	53.7
Urea	population	100x100	126.0
Allantoin	C1	36	11.7
Allantoin	C2	50x50	53.8
Allantoin	C3	50x50	34.0
Allantoin	population	100x100	233.2
Gln/Alla	population	77	45.5
Gln/Pro/Alla/Urea	population	77	45.3
Gln/Pro/Alla	population	77	53.1
Gln/Pro	population	77	45.4
-	FY4	100x100	254.4
