# Supplementary material for: Molecular Specificity, Convergence and Constraint Shape Adaptive Evolution in Nutrient-Poor Environments
Source: PLoS Genet. 2014 Jan 9;10(1):e1004041. doi: 10.1371/journal.pgen.1004041 (PMC3886903; doi:10.1371/journal.pgen.1004041)
Supplement: Table S5 — List of primers used for allele specific PCR genotyping. (PDF) [file pgen.1004041.s016.pdf]

List of primers used for allele specific PCR for clone 3 recovered from the ammonium-limited population

| chromosome | position | WT:MT allele | gene | Common Forward primer | Wild-type reverse primer | Mutant type reverse primer |
|---|---|---|---|---|---|---|
| chr06 | 190932 | T:G | *FAB1* | AAAAACAGCCACACACTTGAGA | AGCTTTACCTGTTTGTTCGA | AGCTTTACCTGTTTGTTCGC |
| chr06 | 96957 | G:A | *GAT1* | TCCTCCTTCGATGACCACTT | GAAGAGGCCGCAAGCATTGC | GAAGAGGCCGCAAGCATTGT |
| chr11 | 117178 | G:A | *LST4* | TGCCATTAGGAGAGGAAGGA | TATTGACATCAGGACGCATTATCC | TATTGACATCAGGACGCATTATCT |
| chr14 | 358507 | G:C | *MEP2* | GTTCGTGCCAATCTGGTC | AGTTAATACAGAACCAATACAAC | AGTTAATACAGAACCAATACAAG |
| chr06 | 74230 | A:C | *RIM15* | CTGGCCACAGAGAAGAATCC | GTTCCACTGTGGAGAGCCA | CTGGCCACAGAGAAGAATCC |
